# A Priori Modeling and Differentiated Parameter Reduction of ECOMC Solar Radiation Pressure Parameters for BeiDou Satellites Based on Driving Attribution

**DOI:** 10.3390/s26196115

**Published:** 2026-09-26

**Authors:** Dongfang Zhao, Houpu Li, Shaofeng Bian

**Affiliations:** 1 Naval University of Engineering, 717 Jiefang Avenue, Qiaokou District, Wuhan 430033, China; 211804020003@home.hpu.edu.cn; 2 School of Geography and Information Engineering, China University of Geosciences (Wuhan), Wuhan 430078, China

**Keywords:** BeiDou Navigation Satellite System (BDS), solar radiation pressure, ECOMC model, a priori constraint, parameter reduction, precise orbit determination

## Abstract

**Highlights:**

**What are the main findings?**

- Which ECOMC solar radiation pressure parameters of BDS satellites can be constrained a priori, and which parameters can be reduced without degrading precise orbit determination.
- The BDS-2 GEO YSIN is almost linear in the solar elevation angle β (constellation-level R2 = 0.988); the BDS-3 IGSO YRAD follows a second-order harmonic in β (R2 = 0.750); and the BDS-3 MEO-CAST DCOS follows a joint model of cosβ dependence plus an eclipse-season offset (*R*^2^ = 0.814; per-satellite IQR = 0.0006). A full-year POD validation shows that indiscriminate ECOMC parameter reduction degrades the within-day fit by a factor of 7.35 relative to free estimation, whereas differentiated partial reduction avoids this degradation.

**What are the implications of the main findings?**

- For BDS satellites, the a priori constraints achieve parameter regularization while preserving orbit-determination accuracy; the resulting orbit-group- and manufacturer-specific prior-constraint and reduction strategies provide an attribution-based basis for parameter handling in BDS POD.

**Abstract:**

Mismodeling of solar radiation pressure (SRP) is a leading error source in BeiDou precise orbit determination (POD), yet the temporal predictability of the empirical SRP parameters remains poor. Using the five-year (2020–2025) daily ECOMC parameter series of 42 BeiDou-2/BeiDou-3 (BDS-2/BDS-3) satellites (67,824 records) and driving attribution, we build orbit-group- and manufacturer-specific prior-constraint and parameter-reduction strategies. The BDS-2 GEO YSIN is almost linear in the solar elevation angle β; the BDS-3 IGSO YRAD follows a second-order harmonic in β; and the BDS-3 MEO-CAST (China Academy of Space Technology) DCOS is well described by a joint cosβ and eclipse-season-offset prior (*R*^2^ = 0.814). These priors are purely empirical statistical models, and the IGSO group contains only three satellites, so transferring these priors to newly launched satellites of the same type carries risk. Full-year 2023 five-scheme POD validation shows the priors are neutral relative to free estimation, whereas indiscriminate parameter reduction degrades the within-day fit by a factor of 7.35; the recommended partial reduction for the BDS-3 IGSO and BDS-3 MEO-SECM (Shanghai Engineering Center for Microsatellites) satellites avoids this degradation. For the CAST group, reduction is not recommended: a control experiment shows the DCOS prior cannot recover the accuracy lost to reduction. Parameter-domain statistics alone cannot guide reduction; orbit-domain POD validation is mandatory.

## 1. Introduction

Solar radiation pressure is the strongest non-conservative perturbing force acting on navigation satellites, and for centimeter-level precise orbit determination (POD) the errors of the SRP model are among the principal factors limiting orbit accuracy. The ECOM family of empirical SRP models proposed by the Center for Orbit Determination in Europe (CODE) [1,2] expands the SRP acceleration in periodic terms along the sun-directed (D), solar-panel-axis (Y), and orbit-normal (B) directions. Because of its simple form and numerical stability, the model has been adopted for years by several analysis centers of the International GNSS Service (IGS). On this basis, Arnold et al. [3] recovered the first-order periodic terms in D and Y and introduced even higher-order terms in D, giving rise to the 13-parameter ECOMC model, which is now widely used in the estimation of precise BeiDou ephemerides.

The 13 ECOMC parameters are usually treated as daily estimates in POD solutions. This practice is flexible, but it raises two issues. First, the parameters are strongly correlated with one another and with the orbital elements [4], so fully unconstrained estimation weakens the solution. Second, a priori information about the parameters has long been unavailable: it has not been established whether the parameters exhibit predictable temporal variations and whether they can be constrained by physical or geometric quantities. On parameter selection, Mao et al. [5] systematically analyzed the parameter-selection strategy of the ECOM model for BeiDou satellites on the basis of overlapping-arc accuracy and proposed a seven-parameter scheme for eclipse and nominal arcs; Li et al. [6] provided significance tests and parameter-reduction recommendations for the ECOMC parameters of BDS-3 satellites. On a priori modeling, Wang et al. [7] constructed an 8-parameter empirical prior model tailored to the static observation geometry and strong parameter correlations of BeiDou GEO satellites, bringing the satellite laser ranging (SLR) validated radial orbit accuracy of the GEO satellites to within 10 cm. Chen et al. [8] introduced fourth- and sixth-order sine terms in D within the ECOM framework and improved the orbit-prediction accuracy of BDS-3 MEO satellites during eclipse seasons. Chen et al. [9] further analyzed the spectral characteristics of the SRP periodic terms in the DYB frame, confirmed that the second-order D term is over-parameterized during eclipse seasons, and showed that removing it reduces the standard deviation of the SLR residuals from 6.2 cm to 3.9 cm for the satellites of the China Academy of Space Technology (CAST) and from 5.7 cm to 3.1 cm for the satellites of the Shanghai Engineering Center for Microsatellites (SECM). In physical SRP modeling, box-wing and adjustable box-wing models constrain SRP through a priori calibration of the optical parameters of the surface panels [10] and thus complement the empirical models. Wang et al. [11] further improved the orbits of BDS-2 IGSO and MEO satellites by compensating for thermal-radiation-pressure effects, and Liu et al. [12] quantified the impact of ECOM SRP model choices on multi-GNSS ultra-rapid orbit determination.

Existing studies either ask which parameters are worth estimating freely, judged by parameter significance or orbit-determination metrics, or adapt the model structure to a specific orbit group; studies that take the full constellation and its long-term parameter series as their object of study and guide a priori modeling from the driving physics of the parameter variations remain rare. This paper builds on a driving-attribution analysis of the five-year (2020–2025) daily ECOMC parameter series of 42 BDS-2/BDS-3 satellites. It proposes a two-criterion screening rule for candidate parameters that measures signal strength by the group-median independent explained variance and inter-satellite consistency by the interquartile range, constructs orbit-group- and manufacturer-specific a priori models of the SRP parameters together with a differentiated parameter-reduction strategy, and evaluates these strategies through a full-year five-scheme POD validation. The present paper is self-contained in the model formulation and data definitions: the estimation method and the quality control of the daily parameter series are summarized in Section 2, and only the attribution conclusions directly relevant to a priori modeling are reviewed here, without duplicating the complete attribution study, the focus being model construction, error budget, and usage recommendations. The remainder of this paper is organized as follows. Section 2 introduces the data, an outline of the attribution method, and the definition of the geometric quantities. Section 3 presents the two-criterion screening rule for a priori modeling and its Bayesian implementation. Section 4 constructs the prior models for the three orbit groups and gives their error budgets. Section 5 presents differentiated parameter sets and a priori constraint strategies by orbit group and manufacturer. Section 6 discusses the POD application prospects, the physical mechanism, and the limitations. Section 7 concludes the paper.

One methodological gap of these existing strategies should be made explicit, because it motivates the design of this paper. Parameter selection has so far been judged either in the parameter domain, by the statistical significance or the attributable structure of the parameter series, or in the orbit domain, by orbit-fit, overlap, prediction, and SLR metrics, and conclusions drawn in one domain have been carried over to the other without verification. The two domains are not equivalent: in the present results, the parameters recommended for reduction by parameter-domain evidence still absorb genuine, unmodeled signals, and removing them from the estimated set (deleting their columns from the design matrix) degrades the orbit solution by a factor of up to 7.35 in the within-day fit, whereas a priori constraints that appear weak in the parameter domain prove neutral and lossless in the orbit domain. Parameter-domain attribution therefore cannot by itself certify a reduction strategy, and orbit-domain validation cannot by itself explain why a strategy succeeds or fails. This paper bridges the two domains by coupling the attribution screening with a full-year five-scheme POD validation, so that every recommended parameter set is confirmed at the orbit level before a recommendation is made.

## 2. Data and Methods

### 2.1. Daily ECOMC Parameter Series

In the ECOMC model adopted throughout this study [3], the SRP acceleration is decomposed in the satellite-oriented DYB frame as D(*u*)·*e_D_* + Y(*u*)·*e_Y_* + B(*u*)·*e_B_*, where *e_D_*, *e_Y_*, and *e_B_* are the unit vectors along the sun-directed (D), solar-panel-axis (Y), and orbit-normal (B) directions and *u* is the argument of latitude of the satellite measured from the Sun; the three component functions are truncated Fourier series in *u*:

(1) $$\begin{array}{c} D(u)=D_{\mathrm{RAD}}+D_{\mathrm{COS}}\cos u+D_{\mathrm{SIN}}\sin u+D_{2\mathrm{CS}}\cos2u+D_{2\mathrm{SN}}\sin2u+D_{4\mathrm{CS}}\cos4u+D_{4\mathrm{SN}}\sin4u\\ Y(u)=Y_{\mathrm{RAD}}+Y_{\mathrm{COS}}\cos u+Y_{\mathrm{SIN}}\sin u\\ B(u)=B_{\mathrm{RAD}}+B_{\mathrm{COS}}\cos u+B_{\mathrm{SIN}}\sin u \end{array}$$

Equation (1) contains the 13 estimated parameters: the constant terms along the D, Y, and B directions (DRAD, YRAD, BRAD), the first-order cosine and sine terms (DCOS, DSIN, YCOS, YSIN, BCOS, BSIN), and the second- and fourth-order cosine and sine terms in D (D2CS, D2SN, D4CS, D4SN) [3]; all parameter values in this paper are expressed in units of 10^−7^ m/s^2^.

The data consist of the daily series of the 13 ECOMC SRP parameters of 42 BDS-2/BDS-3 satellites [13], spanning day of year (DOY) 362 of 2020 to DOY 364 of 2025—67,824 records in total, all sampled at GPST 12:00. The parameter series were obtained by dynamic orbit fitting [14] in which the precise ephemerides of the Multi-GNSS Experiment (MGEX) analysis center of Wuhan University served as pseudo-observations [15] (SP3 files, 5 min sampling); the BDS-3 precise orbit and clock products of the same center [16] and the multi-GNSS MGEX products [17] were used for independent cross-checking of the fitted series. All computations in this study are based on one-day arc fits, for two reasons: first, the ECOMC parameters of the IGS analysis centers are routinely estimated on a daily basis, so one-day arc fits keep the present series consistent with the standard product practice; second, this study investigates the day-to-day temporal variation of the parameters, and multi-day arcs would average out exactly the daily signal of interest. The satellites are divided into six groups by orbit type and manufacturer (Table 1). For each satellite and each day, the equations of motion were integrated numerically over the one-day arc, and the six initial state elements together with the 13 ECOMC parameters (19 parameters in total) were estimated in a least-squares adjustment that minimizes the residuals of the integrated trajectory against the pseudo-observed positions. Each daily fit delivers one set of parameter estimates referred to the GPST 12:00 epoch of that day. These fits were performed with the ORBFIT orbit-fitting module of GAMIT/GLOBK (release 10.76), with the ECOMC solar radiation pressure model and the EGM2008 Earth gravity field model in the force model. The assignment of the 42 satellites to the six groups was verified by cross-checking three independent pieces of evidence: the satellite-type flags recorded in the daily solution files, the semimajor axes and inclinations computed independently from the fitted state vectors, and the published PRN–SVN correspondence, which are fully consistent with one another. Satellite × epoch combinations without a valid daily solution (about 4.5% of the full grid, detailed below) are left as gaps in the series. The five-scheme POD validation was computed separately with the in-house orbit-determination platform (Section 6). The generation of the parameter series (the ORBFIT module of GAMIT/GLOBK) and its orbit-level validation (in-house platform) therefore rest on two separate software chains.

As for data coverage, the five years contain 1691 valid daily-solution epochs, and about 4.5% of the satellite × epoch records are missing, concentrated mainly in several periods of 2022 and 2025. Absences in the BDS-2 GEO group increase from 2023 to 2025; C46 of the 3M-CAST group is largely absent from 2024 onward and completely absent in 2025. The number of valid epochs per satellite ranges from 1134 to 1689. Missing epochs have only a limited effect on the constellation-level combined estimation, and a minimum window sample size (*n* ≥ 100) is imposed in the sliding-window estimation.

**Figure 1:**
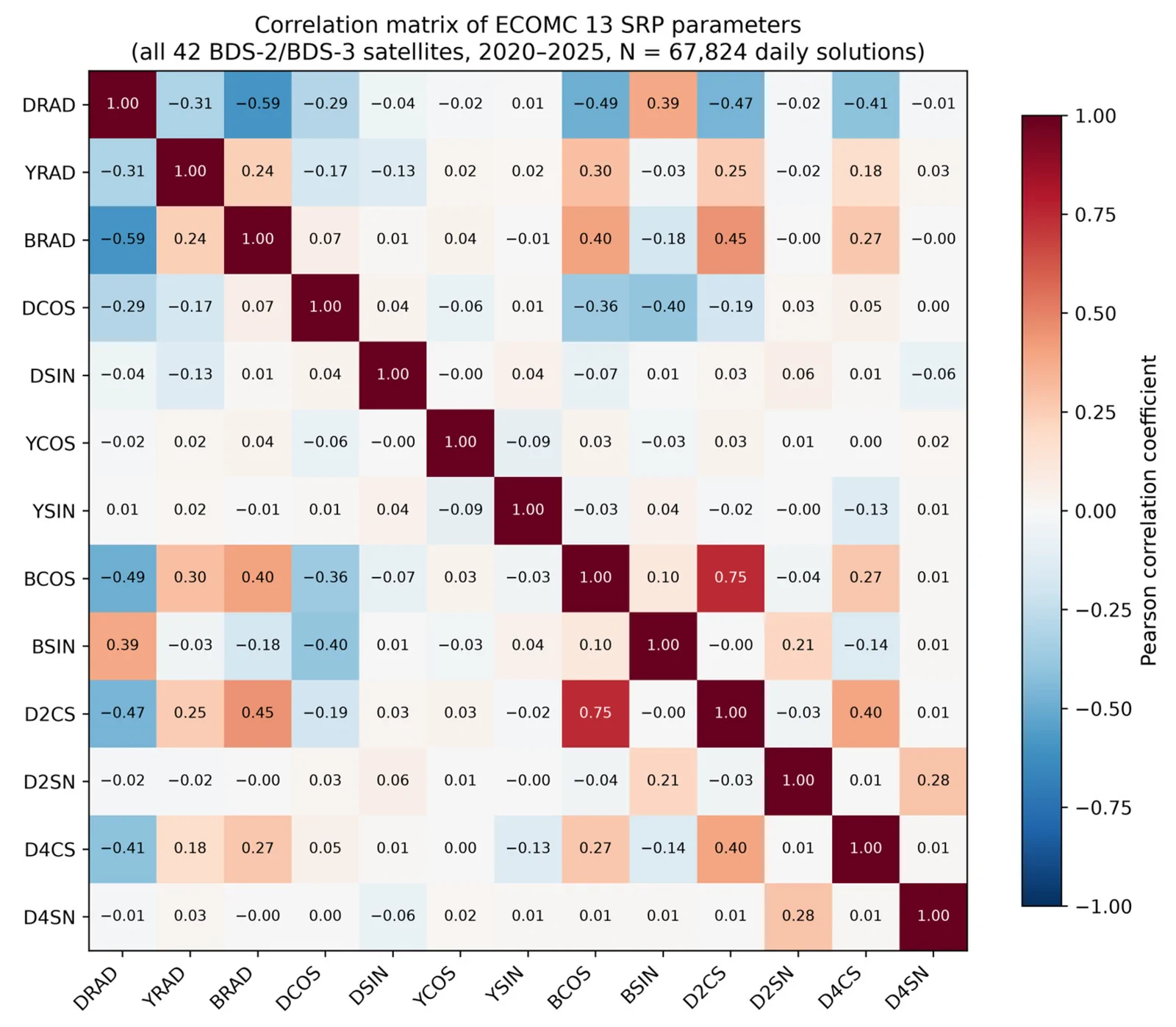
Pairwise Pearson correlations among the 13 ECOMC daily parameter estimates over the full constellation (2020–2025). Missing epochs are excluded pairwise.

The correlations among the 13 estimated parameters were quantified directly from the daily series: pairwise Pearson correlation coefficients were computed within each group and over the full constellation, with missing epochs excluded pairwise. The strongest pair of each group is listed in Table 2, and the full-constellation correlation matrix is shown in Figure 1. Over the full constellation, the strongest correlations are BCOS–D2CS (r = 0.750), DRAD–BRAD (r = −0.590), and DRAD–BCOS (r = −0.491), all involving D- and B-axis terms of constant or low harmonic order. The strongest pair differs from group to group (Table 2), the largest |r| ranging from 0.49 (2M) to 0.78 (3M-SECM), so the strong-correlation structure is not a constellation-wide fixed pattern. A common feature nevertheless exists: the leading correlations of every group involve D- or B-axis terms of the same or adjacent harmonic order, which is consistent with the over-parameterized nature of the ECOMC model, in which constant and periodic terms along one direction can partly absorb each other on long time scales. These results agree qualitatively with the GPS study of Bae and Hong [4], who found the ECOM parameters to be generally correlated, with the correlation pattern depending on the satellite block type and the orbital plane; the present BDS results likewise vary with orbit type and manufacturer, which supports the group-wise treatment adopted in this paper.

### 2.2. Outline of the Driving-Attribution Method

The essentials of the attribution method are as follows. For each satellite and each parameter, a full model y = c0 + cβ·β + cE·E + ct·t + ε is fitted to the five-year daily series, where β is the signed solar elevation angle, E the 0/1 eclipse-season flag, and t the number of years since the satellite’s first epoch. The independent explained variance of driver j is defined as $\Delta R_{j}^{2}$ = *R*^2^_full − *R*^2^_(−j), the difference between the explained variance of the full model and that of the model refit with driver j removed; at the group level, signal strength is measured by the per-satellite median of Δ*R*^2^ and inter-satellite consistency by the interquartile range (IQR). The solar activity index F10.7 was identified in the attribution study as a co-trending artifact with respect to DRAD (its independent explained variance does not exceed 1.3%) and is therefore excluded from the feature set; the three-driver formulation with β, E and t is adopted. A full-matrix check shows that the three-driver and the four-driver (including F10.7) formulations differ by no more than 0.02 in the group-median statistics, so no screening or modeling conclusion is affected.

Multicollinearity among the three drivers was examined directly. Among the pairwise Pearson correlations of the three design matrices, corr(β, t) is −0.074, −0.006, and −0.047 for the 2G, 3I, and 3M-CAST groups, respectively, and corr(E, sin*β*) is +0.015, −0.003, and +0.0007 (E is a 0/1 flag defined on |β|, so this correlation is evaluated against sin*β*); all are below 0.08. The variance inflation factors (VIF) of the design matrices do not exceed 1.007 (2G: 1.006; 3I: 1.000; 3M-CAST: 1.003). After further removing the long-term trend t, the group-median changes in the leading-driver Δ*R*^2^ for the three dominant combinations entering the models (YSIN × β (2G), YRAD × β (3I), DCOS × eclipse season (3M-CAST)) do not exceed 0.016 (a relative change of no more than 2.2%). The three drivers are therefore approximately uncorrelated, the attribution ordering is unaffected by collinearity, and the screening and modeling conclusions remain valid.

**Figure 2:**
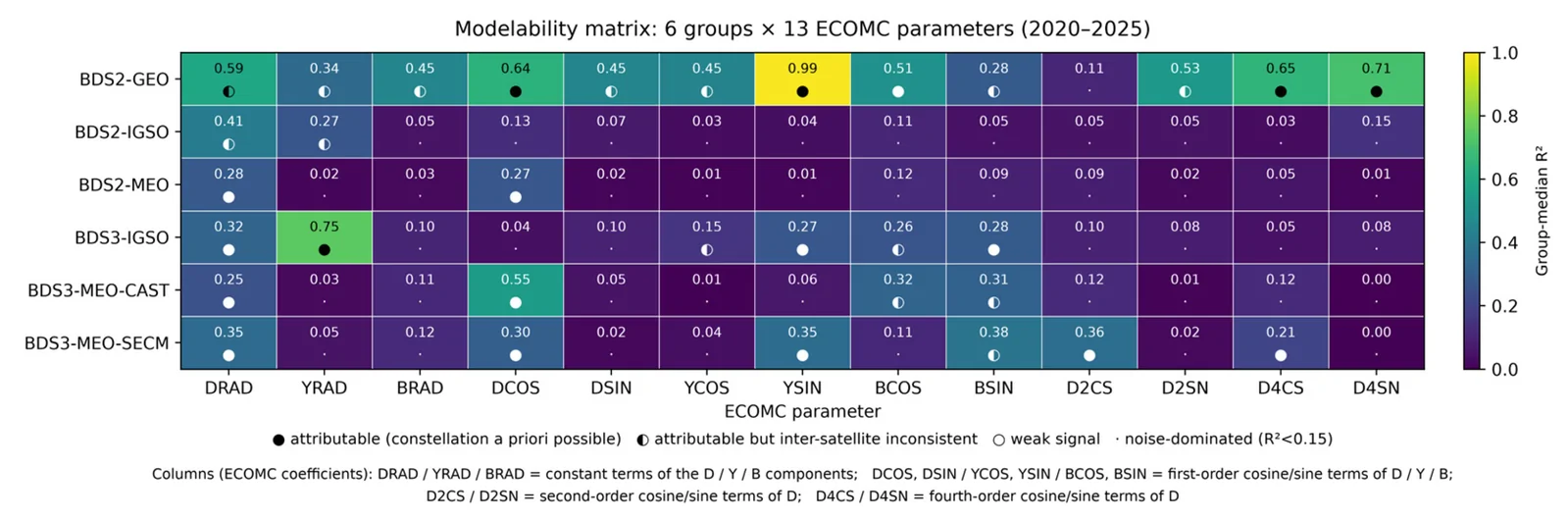
Modelability matrix of the six groups × 13 ECOMC parameters (2020–2025). The color scale shows the group-median *R*^2^ of the full model (β + eclipse season + trend).

An overview of the attribution results for all 78 group × parameter combinations is given in Figure 2. Classified according to the criteria of Section 3, 18 combinations are attributable and within-group consistent, 14 are attributable but inter-satellite inconsistent, and 46 are noise-dominated (group-median *R*^2^_full < 0.15). The three strong-signal combinations directly relevant to a priori modeling are listed in Table 3.

### 2.3. Definition of the Solar Elevation Angle β and the Eclipse Season

The solar elevation angle β is computed as sin*β* = (r☉·h)/(|r☉|·|h|), where h = r × v, with r and v the position and velocity vectors of the satellite in the J2000 inertial frame. The solar position r☉ is taken from the low-precision analytical formula of Montenbruck and Gill [18] (accurate to about 0.01°, a negligible effect for the present residual analysis at the 10^−3^ level). The eclipse-season flag E equals 1 when |β| < βE and 0 otherwise, where βE = arcsin(R⊕/a), R⊕ = 6378.137 km, and a is the semimajor axis, which is computed pointwise from the position and velocity through the vis-viva equation

(2) $$a={\left(\frac{2}{r}-\frac{v^{2}}{\mu }\right)}^{-1}$$

where μ is the geocentric gravitational constant, and r = |r| and v = |v| are the geocentric distance and the velocity magnitude of the satellite at each epoch. The β coverage differs markedly among the groups: the constellation-level β of the 2G group lies in [−23.31°, +23.32°], consistent with the theoretical GEO range; the per-satellite β coverage of the 3I group is ±46.4° (C38), ±78.4° (C39) and ±53.0° (C40); and the group-median βE of the 3M-CAST group is 13.21°. This geometric disparity propagates through all the modeling results below.

## 3. Criteria for a Priori Modeling

A priori models are useful only if two conditions are met: the temporal variation of the parameters contains a systematic component that can be predicted from physical or geometric quantities, and this component shares a consistent form and magnitude across the satellites of a group. Accordingly, two statistics of the attribution matrix serve as the two-criterion screening rule: (i) signal strength, namely a full-model *R*^2^ median of at least 0.15 together with a Δ*R*^2^ median of at least 0.10 for the dominant driver; and (ii) inter-satellite consistency, namely an IQR of the dominant Δ*R*^2^ not exceeding half of its median. Combinations that meet both criteria are regarded as attributable and within-group consistent and may be given a constellation-level prior; those meeting criterion (i) but inconsistent across satellites should be modeled per satellite or left unconstrained; and those with a full-model *R*^2^ median below 0.15 are judged noise-dominated and pass to the reduction discussion. The two criteria serve only as an initial screening. Whether a constellation-level prior is actually established still depends on a cross-satellite transferability test (leave-one-satellite-out cross-validation): the prediction accuracy of the constellation coefficients on the held-out satellite must reach an acceptable level, with no conspicuous single-satellite exception; otherwise, even when the two criteria are met (e.g., the YRAD prior of the 3I group in Section 4.2, whose dominant Δ*R*^2^ has an IQR of 0.213, within half of its median), the model is instead handled as a per-satellite prior. Under this rule, three combinations enter modeling: YSIN × β of the 2G group (Table 3, the strongest and most consistent signal of the full constellation), YRAD × β of the 3I group (strong signal but inter-satellite inconsistent, to be modeled per satellite), and DCOS × eclipse season of the 3M-CAST group (the strongest and most stable signal among all MEO satellites). In addition, D4SN × eclipse season of the 2G group (Δ*R*^2^ median 0.705, IQR 0.185) is strong yet less consistent than the first three and is listed as a second-round prior candidate; it is not modeled in this paper.

Prior information can enter the POD solution by two routes. One is a Bayesian prior, in which a pseudo-observation equation is attached to each parameter using its a priori value and a priori standard deviation σ, with the weight taken as 1/*σ*^2^. The other is parameter fixing or random-walk constraints, in which the parameter is fixed to its a priori value or allowed to evolve slowly according to a prescribed process noise. The former route is adopted here: an error budget is established for each prior model and σ is recommended accordingly. σ covers not only the fitting residual but also the temporal fluctuation of the coefficients, so as to avoid over-tight constraints that would absorb real signals. Parameter reduction can be viewed as the extreme case of constraints: noise-dominated parameters are no longer estimated, which is equivalent to fixing them to zero. The selection among the functional forms of each prior model follows the principle of retaining a form only if it delivers a genuine gain: a more complex form must simultaneously achieve a significant improvement in the Bayesian information criterion (ΔBIC < −10) and a substantial increase in the coefficient of determination (Δ*R*^2^ > 0.01), with its generalization capability additionally checked by cross-validation. The numerical thresholds of the screening rule in Section 3 (a full-model *R*^2^ median of 0.15, a Δ*R*^2^ median of 0.10, and an IQR within half of the median) are empirical calibrations made in this study rather than values adopted from the literature; they are set conservatively, so that borderline combinations are passed on to the cross-satellite transferability test instead of being discarded at the screening stage. The robustness of the screening rule is confirmed by a threshold-sensitivity test. When the full-model *R*^2^ threshold is varied between 0.15 and 0.25, the classification of the 78 combinations changes only marginally: 18/14/46, 18/13/47, and 16/13/49 for thresholds of 0.15, 0.20, and 0.25, respectively (counts of attributable and within-group consistent, attributable but inter-satellite inconsistent, and noise-dominated); the strong-signal combinations (per-satellite full-model *R*^2^ median of 0.991 for 2G YSIN and Δ*R*^2^ median of 0.527 for 3M-CAST DCOS) keep their classes. When the threshold is relaxed to 0.10, besides the 8 combinations moving into the “weak-signal” class (including boundary combinations such as 2G D2CS), another 3 combinations (2I DCOS, 2I D4SN, and 3M-CAST D4CS) move into the “attributable” class. A Benjamini–Hochberg correction applied to the 78 dominant Δ*R*^2^ values (q = 0.05) shows 77 of 78 combinations significant, the only exception being 3M-CAST D4SN (q = 0.204). Note that the strong autocorrelation of the daily series systematically biases the *p* values of the significance tests downward; the effect-size thresholds (Δ*R*^2^ median and IQR) are therefore used as the primary criterion, with significance testing serving only as a supplement.

## 4. Orbit-Group-Specific a Priori Models

### 4.1. A Priori Model of YSIN for the BDS-2 GEO Group

The sample consists of the five-year daily YSIN series of the five 2G satellites (C01–C05), 7143 records in total. The attribution analysis shows that YSIN is almost perfectly coupled with the solar elevation angle β, so the baseline model takes the linear form

*Y*_SIN_ = *a*sin*β* + *b* (3)

The per-satellite least-squares fitting results for the five satellites are listed in Table 4: the correlation coefficient r ranges from 0.9927 to 0.9956 and the coefficient of determination *R*^2^ from 0.9855 to 0.9911; the per-satellite slopes scatter between 0.0893 and 0.0927 (a relative inter-satellite spread of about ±2%), and the intercepts between −0.0001 and +0.0009. Inter-satellite differences are present but far smaller than the signal amplitude, so the group can be modeled with a common set of coefficients. The constellation-level coefficients were solved by two schemes—a combined least-squares fit over the five satellites and per-satellite inverse-variance weighting (weight 1/σ_coef^2^, which downweights the noisier satellites). The two solutions differ by less than 0.1σ; the latter is adopted as the prior:

*Y*_SIN_ = 0.091604sin*β* + 0.000548 (4)

**Figure 3:**
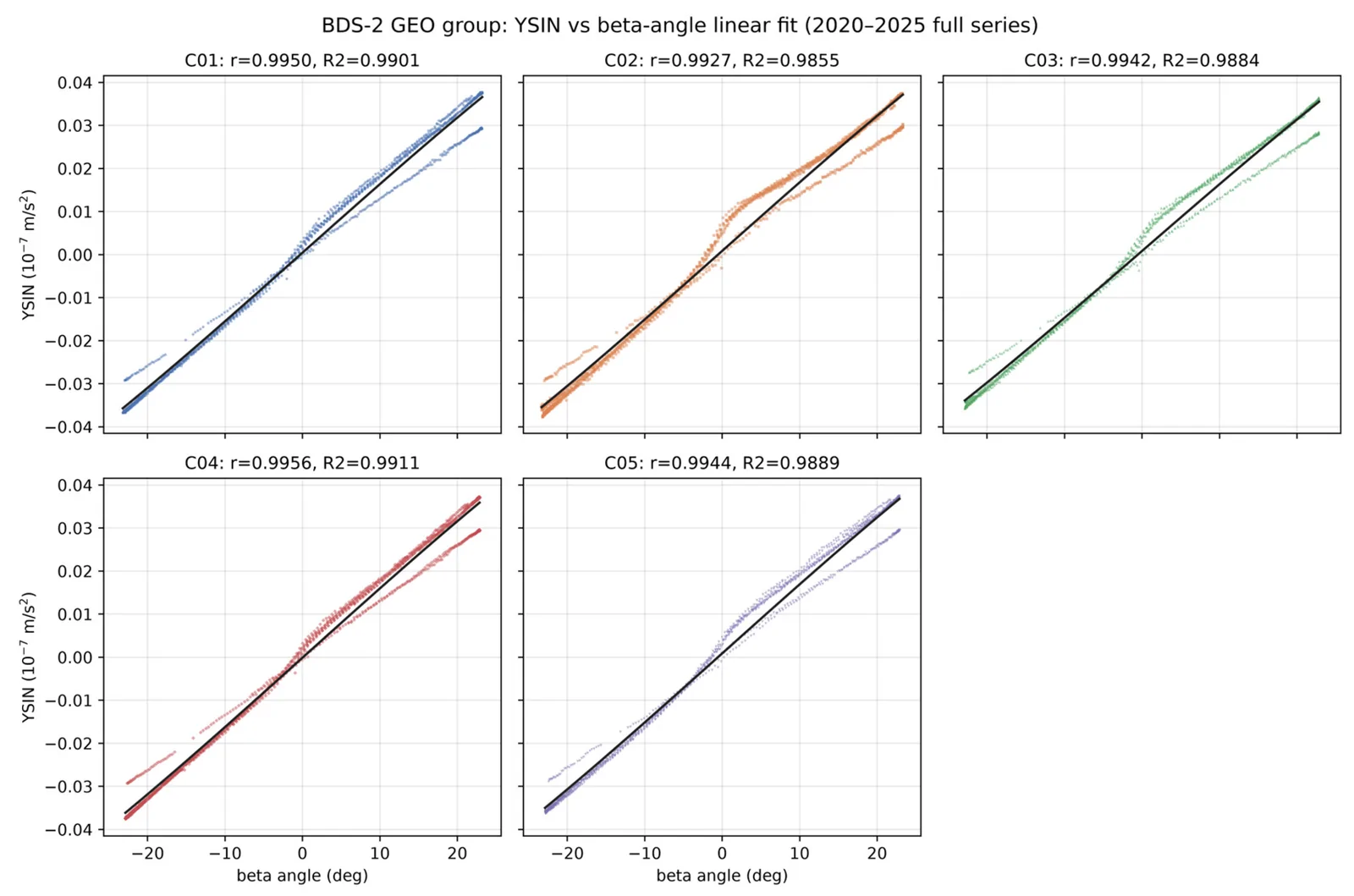
Scatter of YSIN versus sin*β* for the BDS–2 GEO group with the fitted linear a priori model. In each panel, the colored symbols denote the daily YSIN estimates of the individual satellite (C01: blue circles; C02: orange squares; C03: green upward-pointing triangles; C04: red diamonds; C05: purple downward-pointing triangles), and the solid dark line denotes the fitted linear a priori model.

Here the intercept 0.000548 is the inverse-variance-weighted constellation-level OLS intercept b (the constellation-level row of Table 4, re-estimated under heteroskedasticity- and autocorrelation-consistent (HAC) weights, gives 0.000565, a difference of less than 0.1σ; Equation (4) retains the OLS basis so that its intercept and slope come from the same estimator); σa and σb denote the ordinary least-squares (OLS) standard errors (0.000114 and 0.000031); the constellation-level *R*^2^ = 0.988 and the prior residual σ = 2.7 × 10^−3^. Because the daily residuals are autocorrelated with a lag-1 correlation of ρ ≈ 0.99, the Newey–West (Bartlett, m = 20) standard errors are taken as the reference: the per-satellite σa inflates to 0.00118–0.00126 (4.2–5.2 times the OLS value), and the constellation-level (inverse-variance-weighted) σa is 0.000548 (numerically coincident with the intercept b after rounding; the two quantities are unrelated, with unrounded values b = 0.00054807 and σa = 0.00054820). The fit is shown in Figure 3.

**Figure 4:**
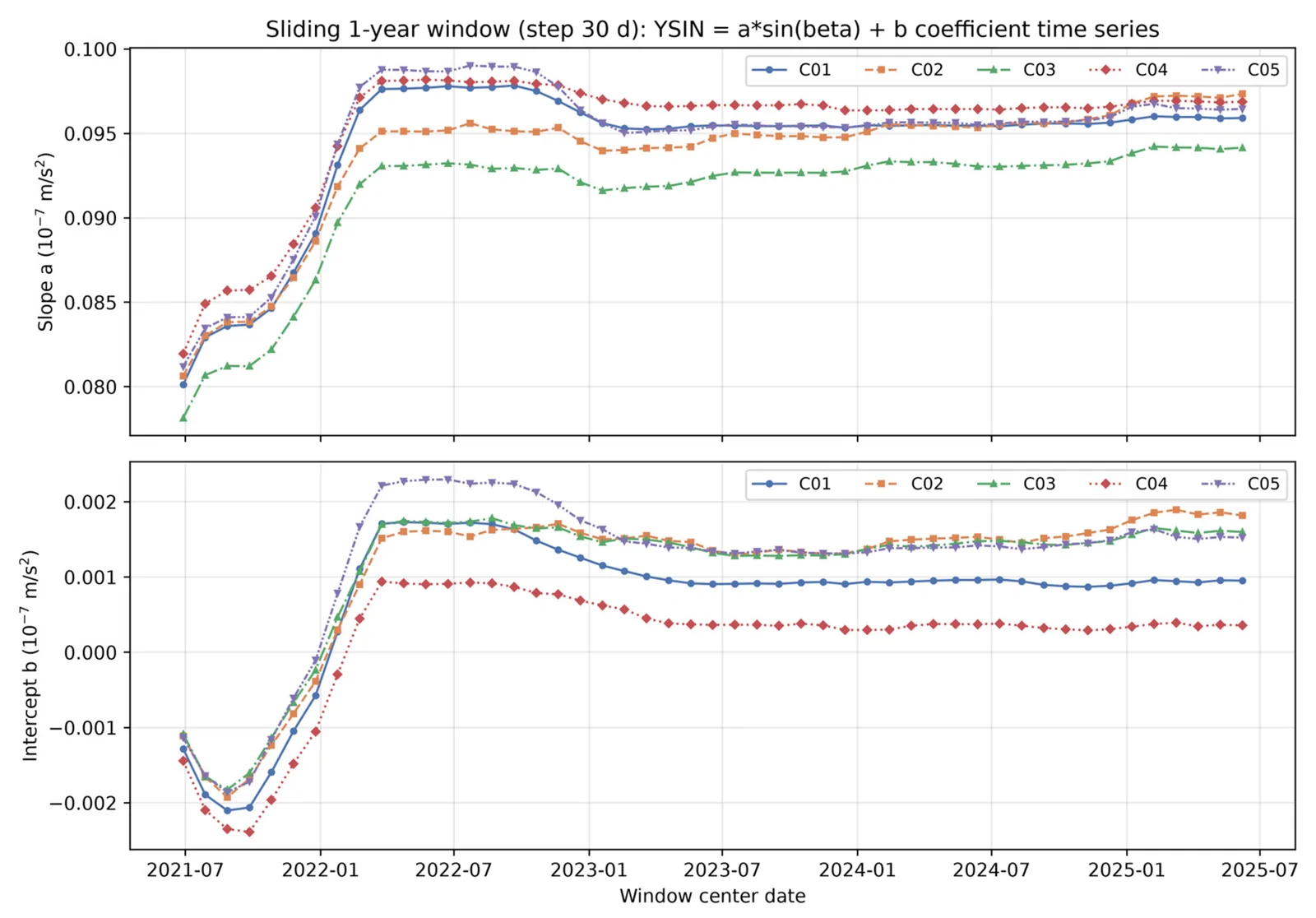
Time series of the sliding-window coefficients of the YSIN prior model (one-year window, step 30 d).

The temporal stability of the coefficients was tested by re-estimation over a sliding one-year window (step 30 d, 49 windows per satellite). The results (right half of Table 4, Figure 4) show that the coefficient of variation CV(a) of the window-to-window slope ranges from 4.13% to 4.57% across the five satellites, with a minimum within-window *R*^2^ of no less than 0.985, so the constant-coefficient assumption holds to first order over the five years. However, the coefficient series reveal a quasi-step rise in coefficient a common to all five satellites from late 2021 to early 2022: with 1 January 2022 taken as a fixed, non-data-driven split point, a ranges from 0.078 to 0.082 before 2022 and from 0.093 to 0.097 afterward, i.e., Δa ≈ 0.015, a relative rise of about 18–19%. The rise is significant under the Newey–West framework: the HAC (m = 20) 95% confidence intervals of the two segments are separated, with z between 7.2 and 8.8; under a long-range robust bound based on AR(1), z drops to 2.2–4.4, C02 being the weakest (z = 2.24; after the AR(1) correction the effective sample size of the segment difference is only about 2, so the 5% level is not reached under that bound), and C04 becoming borderline. A breakpoint scan shows that the two-segment difference actually starts to grow from around mid-2021 and peaks in August–September 2021 (per-satellite z ≈ 23–61); the rise is therefore a transition band spanning roughly half a year rather than an instantaneous step, and is characterized here as a “quasi-step”. Overall, the change is structural rather than pure noise, plausibly related to changes in the in-orbit state of the satellite platforms or in the ephemeris processing strategy; no attribution is attempted here. In practice, piecewise coefficients are not introduced; instead, the temporal fluctuation is absorbed into the prior σ (see the error budget below), retaining the constant-coefficient model at the price of loosened constraints. The piecewise-coefficient and loosened-σ schemes are kept as alternatives to be tested in the follow-up POD validation. If that validation shows the prior to be sensitive to the bias, piecewise coefficients before and after 2022 can be enabled.

Although |β| of the GEO satellites does not exceed 23.5°, such a small range does not guarantee that the linear form is adequate. Two high-|β| corrections were tested on top of Equation (3): adding a sin^3^*β* term (form S3) and a continuous piecewise-linear form with knots at sin*β* = ±sin 15° (form PL). S3 improves the residual σ in the high-β interval by only 0.4% (in none of the six fits, five per-satellite plus the constellation-level fit, does the improvement reach the 5% required for retaining an additional term), whereas PL improves it by 4.7% (only C02, C03, and C05 meet the criterion, and the combined constellation-level fit does not). Although the sin^3^*β* coefficient is statistically significant (|t| ≈ 5–18), the practical gain is negligible: statistical significance does not translate into a useful improvement. Following the principle of retaining a term only when it brings a real gain, the prior keeps the linear form of Equation (3).

The error budget of the prior model is tabulated by |β| interval in Table 5. The prior residual σ shows a shallow U-shape against |β|, higher at both ends and lower in the middle, rising from 2.1 × 10^−3^ in the 10–15° interval to 2.9 × 10^−3^ in the high-β end (20–23.5°). After including the temporal fluctuation of the coefficients (σa = 0.00413, the constellation-level average of the 1σ between-window slope fluctuation of the five satellites; this quantity already contains the effect of the quasi-step above and differs in scope from the HAC standard errors) and combining it as σ = √(σ_interval^2^ + (σa·sin|β|max)^2^), a Bayesian prior σ of 2.2 × 10^−3^–3.3 × 10^−3^ is recommended by interval, or 3.1 × 10^−3^ overall (corresponding to a weight of about 1.0 × 10^5^ in coefficient units, i.e., about 1.0 × 10^19^ in SI units). The weight in the high-|β| interval is about half that in the mid-to-low intervals, so the weight should be set separately by interval in POD. Two caveats are noted. (1) The prior residuals exhibit a visible clustered structure against β, pointing to a secondary modulator beyond β (e.g., satellite attitude mode, thermal deformation) that is not included in the model. (2) The recommended weights are based on empirical residuals and should ultimately be back-calibrated through a residual chi-square test in the POD validation experiments.

### 4.2. A Priori Model of YRAD for the BDS-3 IGSO Group

The association between YRAD and β in the BDS-3 IGSO group is the second strongest after that of the GEO-group YSIN (median Δ*R*^2^ = 0.729; median raw correlation r = +0.863). The SRP behavior of the BDS-3 IGSO satellites has been addressed from the observation-geometry side [19]. Within this dataset, however, the group comprises only three satellites, C38, C39 and C40 (4843 records in total), with markedly different β coverage (Section 2.3). These differences run through all the results of this section and dictate the modeling strategy. All three satellites belong to the operational BDS-3 IGSO series and were manufactured by the China Academy of Space Technology (CAST); they were launched in 2019 as the 44th, 46th and 49th satellites of the BeiDou constellation, and no public source indicates structural differences among them. The inter-satellite differences within this group are therefore confined to the β coverage (Section 2.3) and the in-orbit age rather than to the platform design, and the conclusions of this section are drawn under this same-platform restriction.

Four candidate functional forms are considered, all subject to the acceptance criterion of Section 3: F1, linear (a·sin*β* + b); F2, piecewise linear (knot at β = 0); F3, first-order harmonic; and F4, second-order harmonic. The comparison results are listed in Table 6: neither F2 nor F3 yields a substantial gain over F1, whereas F4 meets the criterion at both the per-satellite and the constellation levels, raising the constellation-level *R*^2^ from 0.639 to 0.750 (ΔBIC = −1757, Δ*R*^2^ = +0.111). The recommended form is

*Y*_RAD_ = *c*_1_sin*β* + *c*_2_cos*β* + *c*_3_sin2*β* + *c*_4_cos2*β* + *c*_0_ (5)

**Figure 5:**
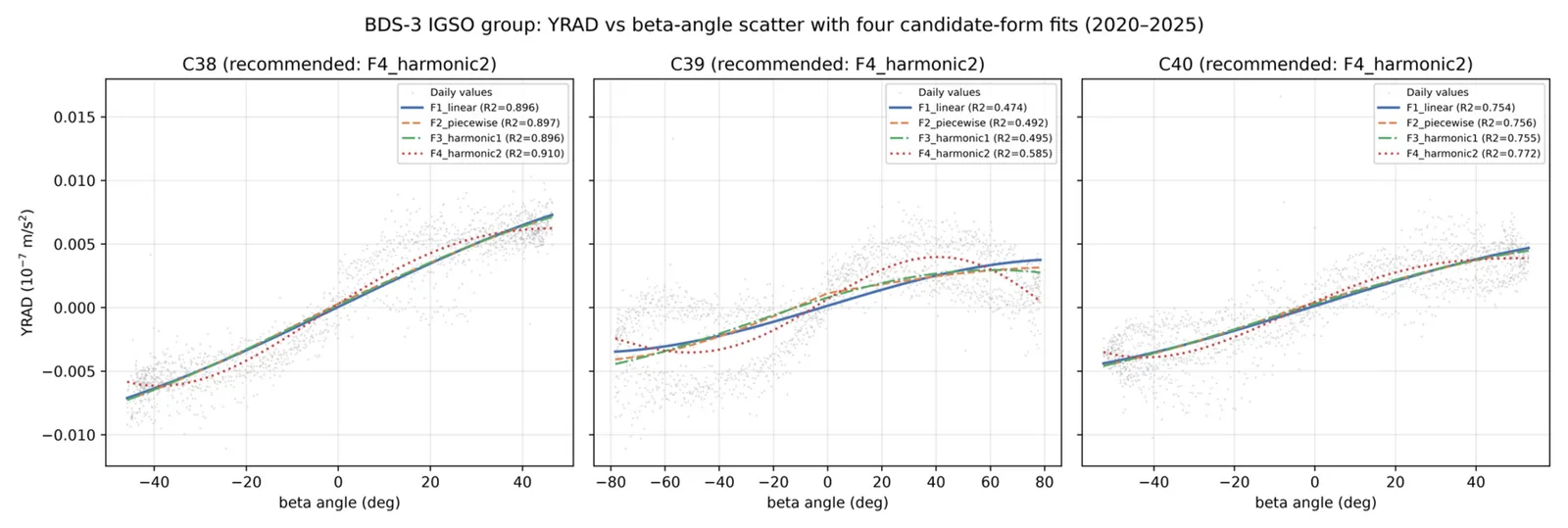
Scatter of YRAD versus β for the BDS-3 IGSO group with fitted curves of the candidate functional forms.

The constellation-level coefficients (full-sample least squares, *n* = 4843) are *c*_1_ = −0.00154 ± 0.00017, *c*_2_ = 0.00255 ± 0.00088, *c*_3_ = 0.00575 ± 0.00013, *c*_4_ = −0.00044 ± 0.00032 and *c*_0_ = −0.00175 ± 0.00060, with a prior residual σ = 2.1 × 10^−3^. The scatter and the fitted curves are shown in Figure 5. A considerable part of the gain in constellation-level *R*^2^ of F4 over F1 comes from absorbing inter-satellite differences: the per-satellite F4 coefficients vary widely (e.g., the cosβ coefficient is −0.0246 for C38 but +0.0034 for C39, and the linear coefficient is −0.0029 for C38 but essentially zero for C39), so the constellation-level coefficients do not correspond to a physical structure common to the group.

**Figure 6:**
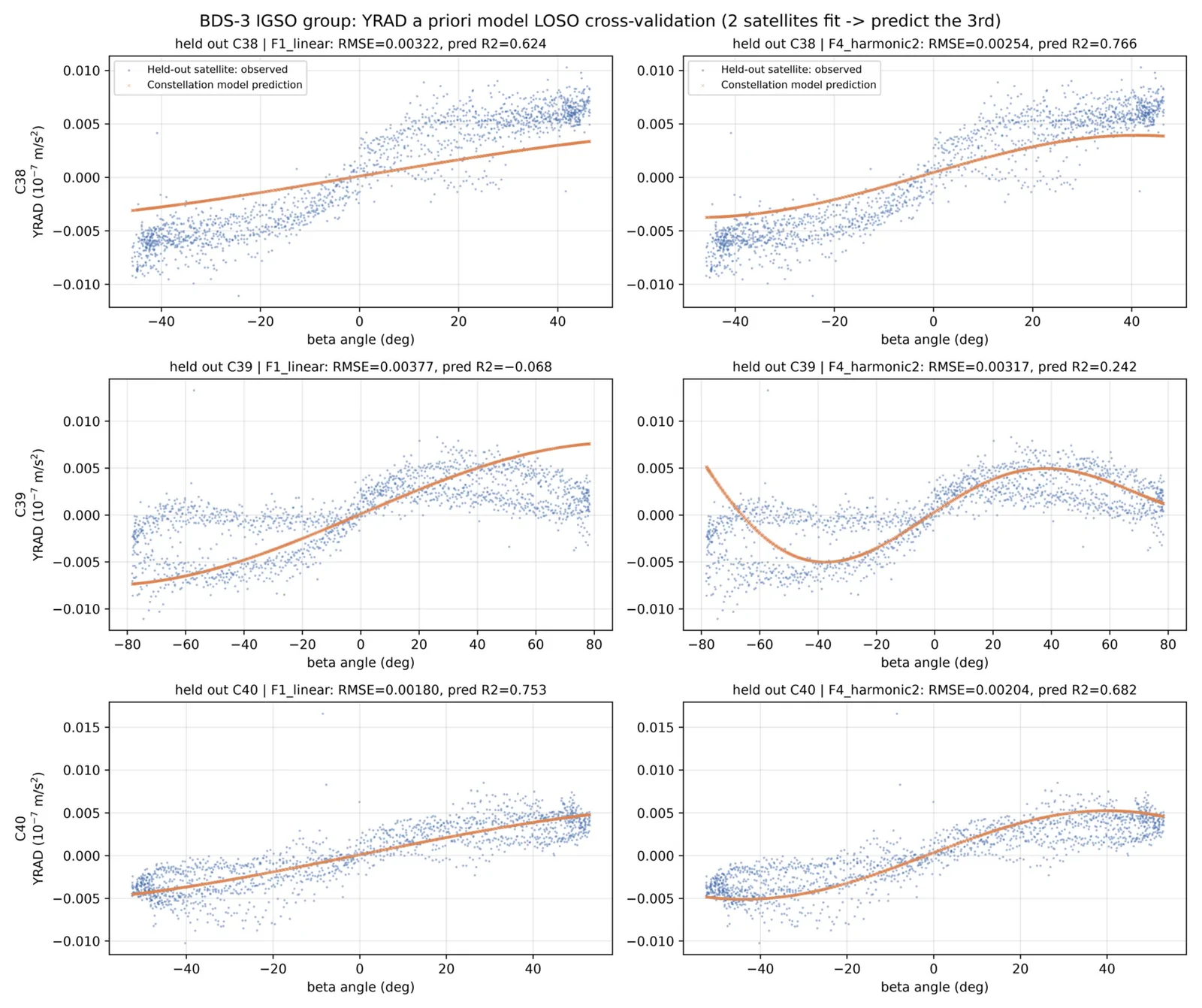
LOSO cross-validation of the YRAD prior model (two satellites fitted, the third predicted; all three satellites are CAST-built BDS-3 IGSO platforms).

The cross-satellite transferability of the constellation-level coefficients is examined by leave-one-satellite-out (LOSO) cross-validation, fitting with two satellites each time and predicting the third (Table 7, Figure 6). In terms of the mean held-out prediction root-mean-square error (RMSE; 0.00259 for F4 against 0.00293 for F1), F4 remains the better form. Its generalization is nonetheless limited and uneven across satellites: the prediction *R*^2^ reaches 0.68–0.77 for C38 and C40 but only 0.24 for C39 (F1 even returns −0.07, i.e., worse than predicting by the satellite’s own mean). C39 differs markedly from the other two in both β coverage (±78°) and coefficient structure, so a two-satellite sample cannot represent it. When C40 is held out, F1 already reaches the per-satellite noise floor (loss factor 1.00, per-satellite fit σ = 0.00180), leaving no room for gain from a more complex form; F4 instead loses 18% because of its extra parameters (loss factor 1.18). The advantage of F4 therefore rests on the constellation-average metric rather than on a uniform per-satellite gain.

As for coefficient stability, the between-window relative fluctuation of the per-satellite linear coefficients over a sliding one-year window is 6.2–17.1% (largest for C39), with no trend-like drift; the temporal stability of the individual coefficients over the five years is acceptable. The inter-satellite coefficient difference, however, reaches a factor of 2.5 (C38 versus C39), which further supports per-satellite modeling as the priority. Accordingly, the usage recommendation is as follows: for a target satellite with a historical parameter series, adopt a per-satellite second-order harmonic prior with σ between 0.0016 and 0.0024 (the per-satellite fit σ values of C38, C39, and C40 are 0.00158, 0.00235, and 0.00174, respectively); for a satellite without historical data, use the constellation-level common coefficients with σ = 0.0021, relaxing to 0.0032 (the upper bound of the held-out prediction RMSE) for satellites with a wide β coverage geometrically similar to C39.

The BDS-3 IGSO group contains only three satellites in this dataset. Although the YRAD–β association is strong in the group-median statistics, the per-satellite analysis shows that both its strength (the independent explained variance Δ*R*^2^ of the β driver, between 0.44 and 0.87 across C38–C40) and the functional coefficients differ markedly across satellites; with only three satellites, the inter-satellite variance component cannot be estimated, and any “manufacturer-level” conclusion is no more than a statement about these three satellites. Under the two-criterion screening of Section 3, the leading-driver IQR of the 3I-group YRAD (0.213) does not exceed half of its median (0.729), so it would have been classified as “attributable and within-group consistent”. The cross-satellite transferability test (LOSO cross-validation, Table 7), however, shows that the constellation-level coefficients generalize only poorly to unseen satellites; following the cross-satellite transferability final ruling in Section 3, the group is instead treated with per-satellite priors. The YRAD–β prior model given in this section should therefore be understood as an empirical summary of the three satellites currently in orbit; its applicability to later satellites of the same group awaits verification on more samples. In POD applications, per-satellite coefficients should be preferred whenever the target satellite has a historical parameter series, and the constellation-level common coefficients serve only as a degraded fallback for satellites without historical data, with the a priori constraints correspondingly relaxed.

### 4.3. Joint cosβ and Eclipse-Season-Offset Model of DCOS for the BDS-3 MEO-CAST Group

**Figure 7:**
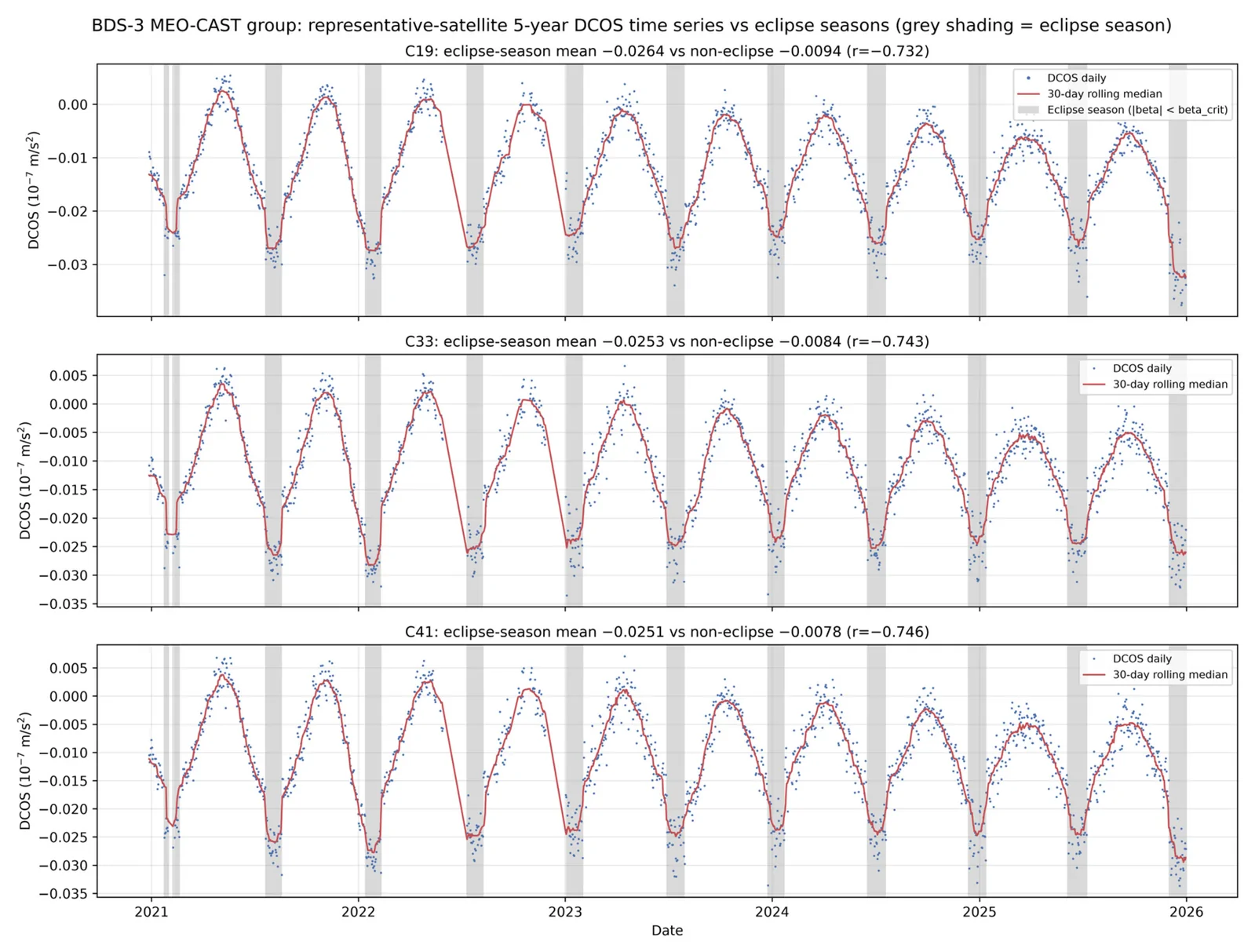
DCOS daily series against eclipse-season intervals (representative satellites C19, C33, C41).

DCOS × eclipse season is the strongest and most consistent attribution signal of the BDS-3 MEO-CAST group (group-median Δ*R*^2^ = 0.527, IQR = 0.051). The sample consists of 22,924 records from the 14 satellites of this group (C46 has been largely absent since 2024, with only 1134 records). Because C46 was completely absent in 2025, its contribution to the constellation-level statistics is reduced; this absence does not affect the piecewise-structure conclusions. A per-satellite statistical verification confirms the attribution: the eclipse-season mean of DCOS (group median −0.0253) differs significantly from the non-eclipse-season value (−0.0078), the group median of the eclipse-season minus non-eclipse-season mean difference is −0.0173, the standardized effect size is −1.89, and the median point-biserial correlation is r = −0.7305 (per-satellite values between −0.746 and −0.510). Within an eclipse season DCOS is systematically shifted negative, and the temporal lows correspond one-to-one to the eclipse-season intervals (Figure 7). The attribution conclusion that the eclipse season is the dominant driver of DCOS is thus independently confirmed.

**Figure 8:**
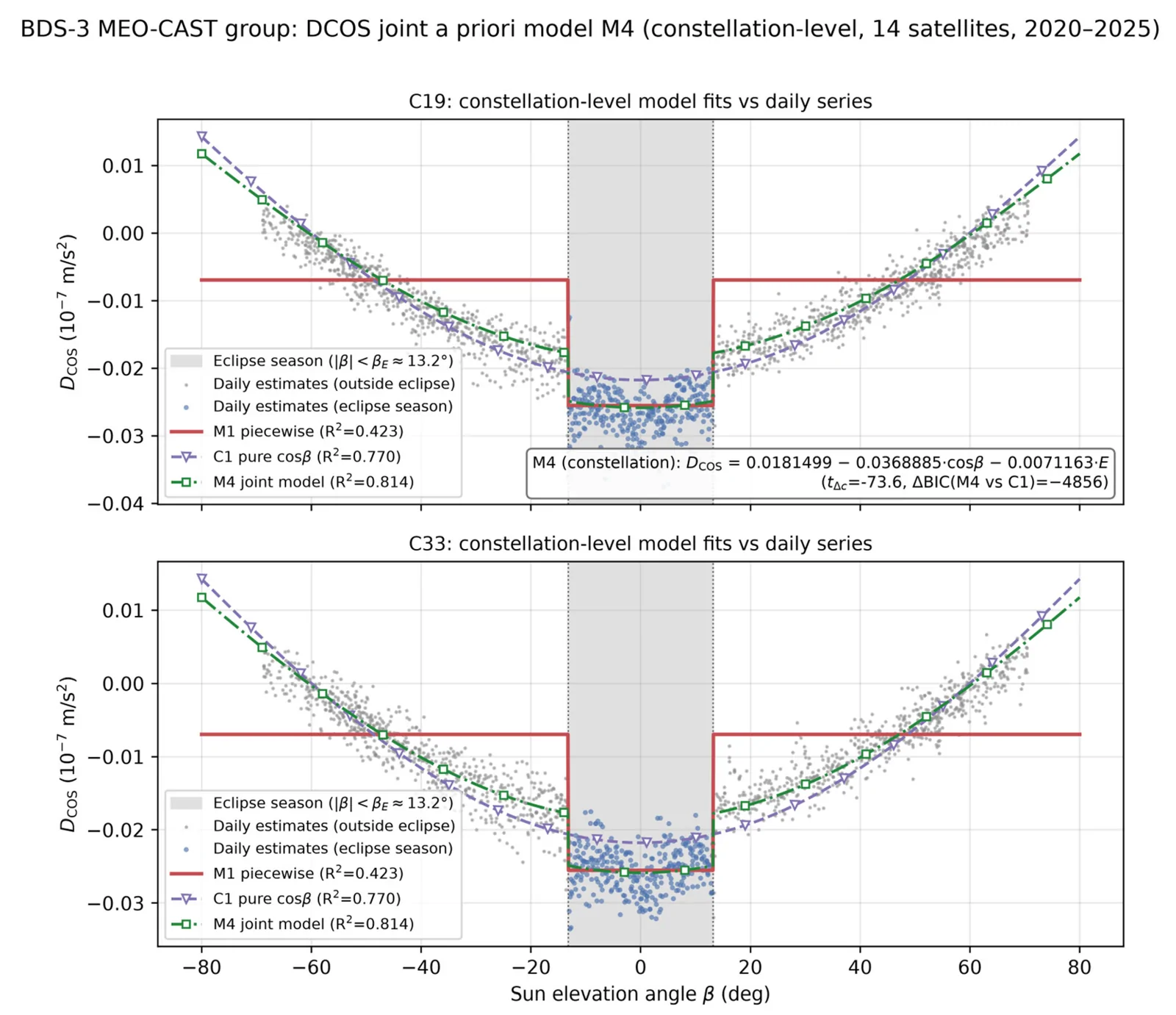
Fit of the three DCOS models to the daily series of the representative satellites C19 and C33: observations (dots), the piecewise-constant model M1 (solid line), the pure cosβ model C1 (dashed line) and the joint model M4 (dash-dotted line); shaded regions denote eclipse seasons.

**Figure 9:**
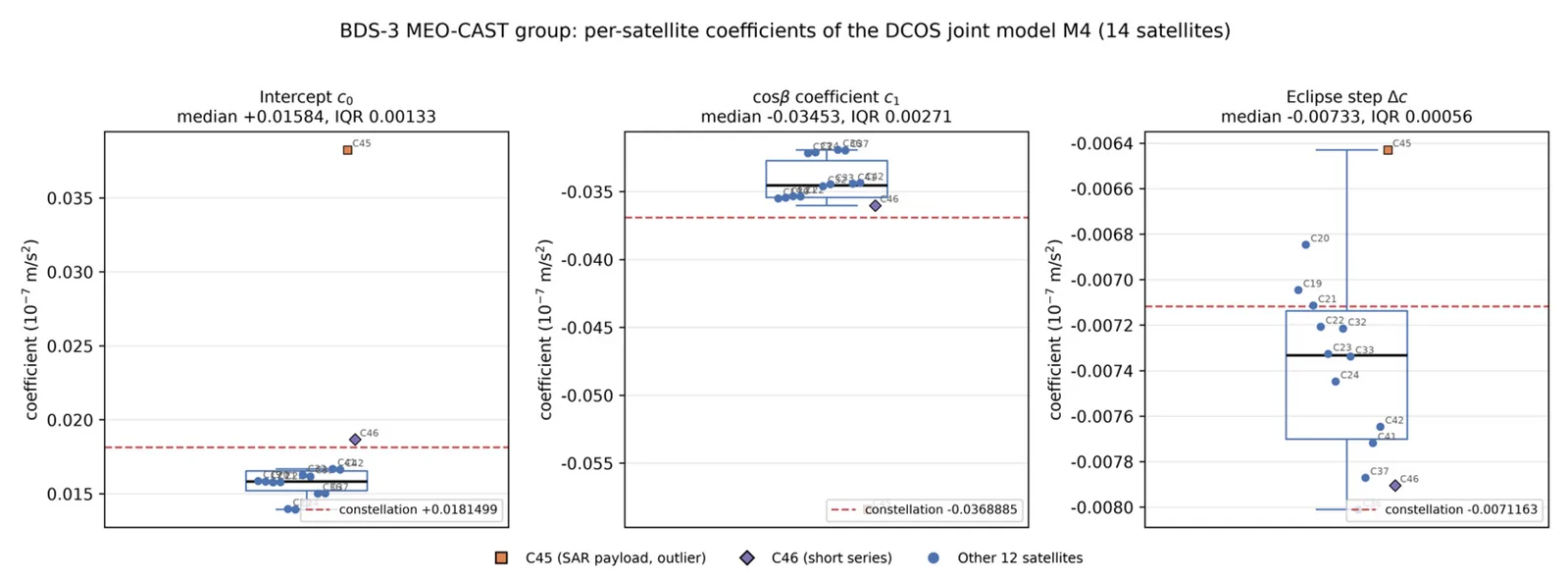
Distribution of the per-satellite coefficients of the DCOS joint model, showing the within-group consistency of Δc and the outliers C45 and C46 (short series).

Three model forms were compared for the constellation-level DCOS prior: M1, an eclipse-season piecewise-constant model; C1, a purely continuous form in cosβ; and M4, a joint model combining the continuous dependence with the eclipse-season offset,

*D*_COS_ = *c*_0_ + *c*_1_cos*β*+Δ*c* · *E* (6)

where E = 1 denotes the eclipse season (|β| < 13.2°) and E = 0 otherwise; c0 and c1 are the intercept and the coefficient of the continuous cosβ dependence, and Δc is the additional eclipse-season offset. Fitted to the 22,924 records of the 14 satellites, the pure cosβ model C1 (M4 with Δc fixed to zero) already reaches a constellation-level *R*^2^ of 0.770 (per-satellite median 0.859), far above the 0.423 of M1 (per-satellite median 0.534), so the continuous dependence is real and dominant. The eclipse-season offset, however, is not absorbed by the continuous term: on top of C1, the joint model M4 still yields a highly significant residual offset Δc = −0.0071 (t = −73.6), raises the constellation-level *R*^2^ to 0.814 (per-satellite median 0.923), and reduces the BIC by 4856 relative to C1. Within an eclipse season, cosβ varies by only 0.026, i.e., 3.4% of its full-year variation range, so no low-order smooth function of cosβ can reproduce the observed eclipse-season step; the continuous term and the eclipse-season offset are therefore not interchangeable, and M4 is adopted as the constellation-level DCOS prior model (Table 8, Figure 8 and Figure 9). The per-satellite residual offsets Δc under M4 are tightly clustered (median −0.00733, IQR 0.0006, a relative dispersion of about 8%), so the within-group consistency conclusion is reinforced under the joint model.

*D*_COS_ = 0.01815 − 0.03689cos*β* − 0.00712*E* (7)

The OLS standard error of the constellation-level Δc is 0.000097. The daily residuals are strongly autocorrelated (the lag-1 autocorrelation of the M1 residuals is ρ ≈ 0.91), so the formal significance is inflated; even if the standard error is inflated by a factor of 2 (the inflation estimated for M1 by a Newey–West re-estimation), the offset remains significant at |t| > 35. The constellation-level RMSE decreases from 0.008227 (M1) and 0.005197 (C1) to 0.004674 (M4). The failure of M1 is now explainable: its piecewise-constant design matrix contains no β-dependent term, so the smooth cosβ dependence (an even function of β, which the linear-in-β attribution design matrix of Section 3 cannot represent either) is absorbed into the step coefficient, and the eclipse-season contrast is inflated to −0.0186, about 2.5 times the genuine offset (per-satellite median −0.0073) isolated by M4. Units: the coefficients and σ are in 10^−7^ m/s^2^; to convert to SI units, σ is multiplied by 10^−7^ and the weight 1/*σ*^2^ by 10^14^ (same convention as in Section 4.1, Table 5).

Two individual cases within the group stand out. C45 carries a search-and-rescue payload; under M4 its fit is the poorest in the group (*R*^2^ = 0.664, RMSE = 0.0112), and its c0 = 0.0383 deviates strongly from the rest of the group (0.0139–0.0187). C46 has a short series (1134 records; *R*^2^ = 0.727). The within-group consistency conclusion rests mainly on the remaining 12 satellites; we therefore recommend excluding C45 from the constellation-level prior and treating it separately.

Error budget: the per-satellite residual RMSE under M4 is 0.00221–0.00266 for the 12 consistent satellites, 0.01120 for C45 and 0.00631 for C46, and the constellation-level RMSE is 0.004674. The a priori σ applied to the CAST DCOS pseudo-observations in the POD solutions is assigned per day and uniformly within the group: over the full year 2023 it takes 32 discrete levels between 0.7194 and 0.7656, with a median of 0.7540 (in the dimensionless coefficient units of the POD prior table; the corresponding physical σ is obtained by multiplying the table value by the satellite-specific F0/M (1.13–1.27 × 10^−7^ m/s^2^)). This σ is constructed from the within-group daily scatter of the POD parameter estimates and is therefore deliberately much looser than the fit residuals above, so the constraint regularizes the solution without forcing it toward the prior mean. Switching the prior mean from M1 to M4 leaves this σ unchanged; only the daily prior mean μ is updated. A full-year 2023 POD re-run with the M4 prior (4955 arcs) confirms that the neutrality of the prior constraint is preserved: all four validation metrics agree with the piecewise-prior results to within 0.24% at the group-median level (Appendix A, Table A1).

In comparison with earlier work, Chen et al. [8] introduced fourth- and sixth-order sine terms in D within the ECOM framework for BDS-3 MEO satellites, improving the root-mean-square (RMS) error of the predicted orbits over deep eclipses from about 7/14/26 cm to about 3/5/12 cm in the radial, cross, and along directions, respectively. That study addressed the D-direction problem in the eclipse period from the model-structure side (by adding higher-order terms). This section shows, from the parameter time-behavior side, that a prior combining the continuous cosβ dependence with an eclipse-season offset on the first-order cosine term in D can absorb the systematic variation of the same physical origin. The two approaches are complementary. Chen et al. [9] defined the eclipse period of BDS-3 MEO satellites as |β| < 12.9°, which is basically consistent with the ≈13.2° used here (the difference stems from their penumbra-onset criterion); Li et al. [20] analyzed the degraded orbit and positioning accuracy of the new-generation BeiDou satellites during Earth eclipse seasons, and Rodriguez-Solano et al. [21] reported the corresponding eclipse-season orbit improvement for GPS. Chen et al. [9] also confirmed that the second-order D term is over-parameterized during the eclipse period, whose removal lowers the eclipse-period SLR residual standard deviation from 6.2 cm to 3.9 cm (CAST) and 5.7 cm to 3.1 cm (SECM). Their conclusion and the present strategy of handling the D-direction parameters separately for eclipse and non-eclipse seasons corroborate each other.

## 5. Differentiated Parameter Sets and a Priori Constraint Strategies by Orbit Group and Manufacturer

**Figure 10:**
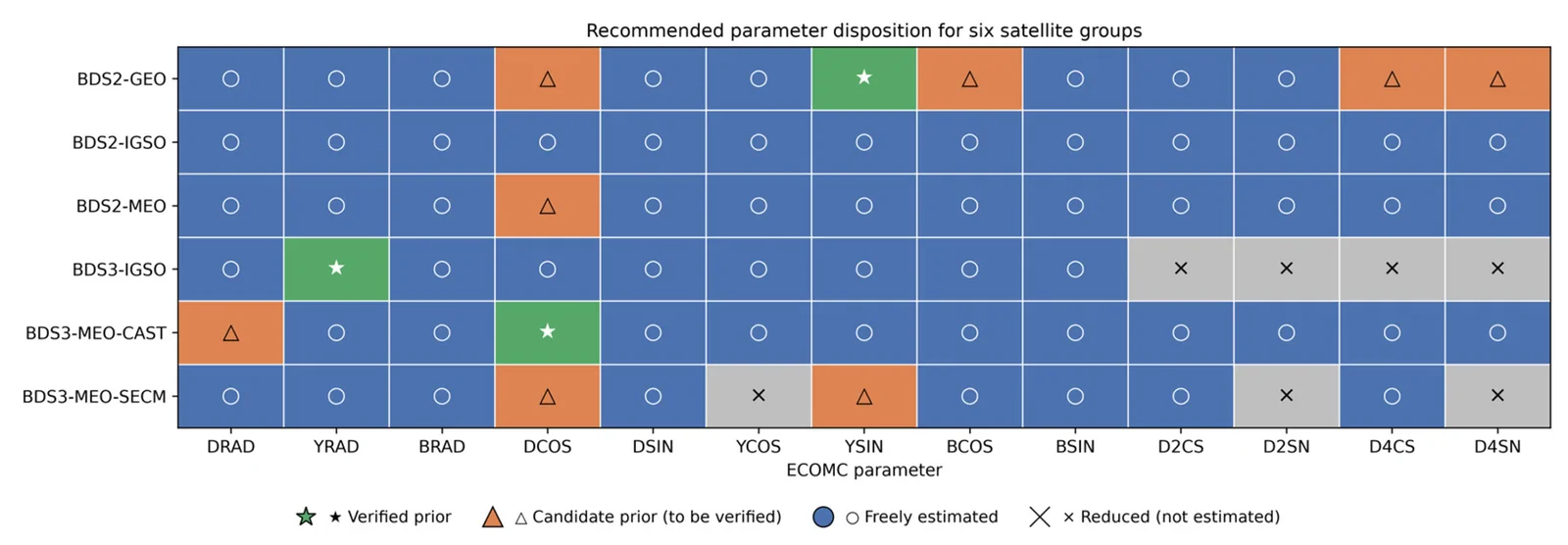
Disposition matrix of the ECOMC parameters for the six groups (★ = verified prior; △ = candidate prior, to be verified; ○ = freely estimated; × = reduced).

Prior modeling covers only the few strong-signal parameters. For the majority of the remaining parameters, the attribution evidence serves the opposite purpose—it shows that they carry no attributable structure and that freely estimating them yields little benefit, which in turn supports reduction. Following the modelability classification of Section 3, recommended parameter sets and a priori constraint schemes are given for each of the six satellite groups (Table 9; the disposition matrix is shown in Figure 10) and compared with parameter-selection strategies in the literature. The five POD validation schemes (A–E) cited in this section are defined together in Table 10 at the beginning of Section 6.1. In summary, reduction is recommended only for the 3I group (to nine parameters) and the 3M-SECM group (to ten parameters); the 2G, 2I, 2M and 3M-CAST groups retain the full 13-parameter set, with the verified a priori constraints applied where available. The three-driver recomputation yields 46 noise-dominated combinations, only one more than the 45 obtained on the four-driver basis that includes F10.7. The difference is a single boundary case, the 2G D2CS (group-median full-model *R*^2^ of 0.108 vs. 0.192), whose independent Δ*R*^2^ stays below 0.06 for all three drivers and which does not affect the conclusion of not reducing this group.

BDS-2 GEO: no reduction. Except for the boundary case D2CS (group-median full-model *R*^2^ of 0.108 on the three-driver basis, below 0.19), the group-median full-model *R*^2^ of the remaining 12 parameters is no lower than 0.19 (the observed minimum within this group, not a screening threshold). The eclipse-season-related higher-order terms carry genuine systematic signals—D4SN × eclipse season (*R*^2^ = 0.705), DCOS × eclipse season (0.507) and D2SN × eclipse season (0.447)—and removing any of them would leave that signal without an absorbing channel and let it contaminate the remaining parameters. All 13 parameters are therefore recommended to be freely estimated for this group, with YSIN constrained by the prior of Section 4.1; DCOS, BCOS, D4CS and D4SN reach the attributable threshold in within-group consistency and are listed as second-round prior candidates.

BDS-2 IGSO and BDS-2 MEO: reduction to ECOMC-7 was originally suggested, with DRAD, YRAD, BRAD, DCOS, DSIN, BCOS, and BSIN as the estimation set. The six dropped parameters (YCOS, YSIN, D2CS, D2SN, D4CS, and D4SN) have parameter-domain group-median full-model *R*^2^ of 0.033–0.147 (2I) and 0.011–0.086 (2M), all below 0.15, so the parameter-domain cost of dropping them appears small; this seven-parameter set also coincides with the empirical strategy of Mao et al. [5]. The E-scheme POD validation, however, shows that this reduced set degrades markedly at the orbit level: it is numerically identical to the indiscriminate reduction (B) (E/B = 1.000), with the within-day fit degrading by a factor of 5.2 (2I) and 6.8 (2M) and the 1-day orbit-prediction extrapolation by a factor of 3.2 and 2.6 relative to fully unconstrained estimation, and no verified prior exists for these two groups to bundle for recovery. Reduction is therefore not recommended for these groups for the time being (restore some parameters or treat them as candidate reductions pending POD validation). The attribution evidence shows that the parameters have no predictable structure, yet the POD validation shows that deleting columns worsens the solution under the weak observation geometry: the two criteria, attribution (parameter domain) and POD (orbit domain), are not equivalent for the weak-geometry groups. Although DRAD × trend reaches the attributable threshold (2I 0.299, 2M 0.154), the trend driver simultaneously absorbs satellite age and processing-strategy drift without a single mechanistic attribution, so it is kept freely estimated without a prior; the 2M DCOS × eclipse season (0.251, IQR 0.086) is listed as a candidate prior, with its functional form and σ to be verified. This verdict differs from the classical ECOMC-7 scheme of Mao et al. [5]; the possible origins of the discrepancy are discussed in the comparison with earlier parameter-selection strategies below.

BDS-3 IGSO: partially reduced to 9 parameters. The four terms D2CS, D2SN, D4CS and D4SN have group-median full-model *R*^2^ of 0.048–0.100, all below 0.15, and are therefore dropped; YCOS and YSIN, which carry attributable β signals (full-model *R*^2^ of 0.154 and 0.270; median Δ*R*^2^ of β of 0.143 and 0.249), are retained for free estimation. The recommended estimation set is ECOMC-7 plus YCOS and YSIN, nine parameters in total. This strategy differs from the ECOMC-8 of Li et al. [6] (which includes D2CS but no first-order Y periodic terms): the measured 3I D2CS is noise-dominated here (group-median full-model *R*^2^ 0.100), and wherever the two differ, the present measured attribution evidence prevails. YRAD is constrained by the per-satellite prior of Section 4.2; given that the group contains only three satellites and the LOSO generalization for C39 is poor, all conclusions await confirmation with an expanded sample.

BDS-3 MEO-CAST: reduction to ECOMC-7 was originally suggested. The six dropped parameters have group-median full-model *R*^2^ of 0.001–0.124, all below 0.15. DCOS is constrained by the joint prior of Section 4.3 (C45 handled separately); BSIN × β (median Δ*R*^2^ 0.278) is inter-satellite inconsistent (IQR 0.240, the β-sensitive B-component differing between C45/C46 and the remaining satellites) and therefore receives no constellation-level prior; DRAD × eclipse season (0.239, IQR 0.042) is listed as a follow-up candidate. The E-scheme POD validation, however, shows that this reduced set is numerically identical to the indiscriminate reduction (B) (E/B = 1.000), degrading by a factor of 7.7 in the within-day fit, 2.9 in the 1-day orbit-prediction extrapolation, 1.96 in SLR off1 and 8.7 in GBM relative to fully unconstrained estimation. Whether the DCOS prior can recover this degradation was tested directly by a control experiment: augmenting the reduced ECOMC-7 scheme with the M4-based DCOS prior (scheme EM4, 4955 full-year arcs) changes the seven metric entries of the CAST group (spanning the four validation metrics) by at most 0.6% relative to E (recovery rate below 1%; per-satellite recovery rates between −1.9% and +2.7%). This confirms that the DCOS prior regularizes the physical plausibility of the estimates rather than compensating the accuracy loss caused by removing parameters, which stems from the deleted higher-order harmonic degrees of freedom and cannot be recovered through a prior on the DCOS term alone. The CAST group is therefore recommended to retain the full 13-parameter set with the DCOS prior applied, and reduction to ECOMC-7 is not recommended.

BDS-3 MEO-SECM: reduced to only 10 parameters. The three terms YCOS, D2SN and D4SN, with group-median full-model *R*^2^ of 0.004–0.042, are dropped; however, YSIN × β (0.354, IQR 0.010), D2CS × eclipse season (0.291) and D4CS × eclipse season (0.204) carry genuine signals, so ECOMC-7 cannot be applied indiscriminately to this group. This point diverges from the strategy of Chen et al. [9] of removing the second-order D term in the eclipse period. That conclusion corroborates the noise-dominated D2CS/D2SN result of the present CAST group, but the eclipse-season signal of the SECM D2CS is genuine, and the “drop-D2” practice cannot be carried over to SECM; D2CS is retained here, and YSIN × β and DCOS × eclipse season are listed as candidate priors (valid only for the eight satellites other than C25/C26; the eclipse-season response of DCOS has the opposite sign to CAST, so the two group models cannot be shared). The E-scheme POD validation shows that this 10-parameter recommended set clearly outperforms the indiscriminate reduction (E/B ratios of 0.57 in the within-day fit, 0.61 in the 1-day extrapolation and 0.56 in GBM), so the recommendation holds; relative to fully unconstrained estimation, however, it still costs a factor of 1.2–3.5 across the four metrics (the loss from dropping YCOS, D2SN and D4SN), an acceptable partial reduction.

The methodological difference between the present strategy and earlier parameter-selection work should be made explicit. Mao et al. [5], Li et al. [6] and Chen et al. [9] judge by parameter significance, orbit-fit/overlap/prediction accuracy or SLR residuals and ask which parameters are worth estimating freely for POD; the present work judges by driving attribution (leave-one-out independent explained variance Δ*R*^2^) and within-group consistency (IQR) and asks which parameters vary predictably with geometric–temporal quantities and are therefore suited to a priori constraints, and which parameters, lacking any attributable structure, should be reduced. The two sets of criteria agree for most groups (ECOMC-7 for BDS-2 IGSO/MEO and BDS-3 MEO-CAST) and diverge for GEO (higher-order terms carry genuine signals, so no reduction) and SECM (D2CS/D4CS carry eclipse-season signals and are partially retained), where the present measured attribution evidence prevails. Four concrete differences between the two studies qualify the divergence from Mao et al. [5] on the BDS-2 IGSO/MEO groups. First, the recommendation of Mao et al. [5] is itself segmented: ECOM-7 is advised only for the eclipse and zero-bias arcs and ECOM-5 for the remaining normal arcs, so their scheme is not a year-round seven-parameter solution of the uniform kind tested in the present POD validation. Second, the parameterization and the baseline differ: their screening compares the 5-, 7- and 9-parameter sets of the ECOM1 family (the seven-parameter set comprises D0, Y0, X0, XC, XS, DC and DS), whereas the present reduction deletes six columns from the 13-parameter ECOMC set, and the two estimation spaces therefore absorb unmodeled signals differently. Third, their own validation already indicates that the enlarged set is harmful on the normal arcs: for C08 the SLR RMS on the normal arc degrades from 0.06 m to 0.12 m when the seven-parameter set replaces the five-parameter set, and the radial overlap precision on the normal arcs degrades by 38–49%, a direction consistent with the full-year degradation measured in this work. Fourth, the observation era and the parameter-series length differ: their analysis rests on three-day arc fits spanning 2013–2016 (3.2 years), whereas the present series consists of daily arcs over 2020–2025, with the BDS-2 satellites almost a decade older. The divergence is therefore reported here as a measured discrepancy rather than a refutation of their scheme.

In addition, a subtype split not previously reported is found within the SECM group: the Δ*R*^2^ values of C25 and C26 are close to zero for YSIN, DCOS, and D2CS, in contrast to the high consistency among the other eight satellites (per-satellite r ≈ +0.59 to +0.60 for YSIN × β); for BSIN × β the per-satellite correlations even split in sign, ranging from −0.64 to +0.84. This suggests possible differences in payload or attitude control within the SECM platform; the attribution of this subtype and subtype-level modeling are left to further work.

## 6. Discussion

### 6.1. Prospects for Applying the a Priori Models in POD

All three a priori models in this study are given as an a priori value plus an interval-dependent a priori σ. They can be introduced directly into POD as pseudo-observation equations with weights 1/*σ*^2^. The σ already incorporates five-year temporal variations in the coefficients (for the GEO group, including the quasi-step enlargement around the end of 2021 to early 2022) and the reduction in effective sample size due to autocorrelation, so the constraints are not so tight as to absorb real signals. Order reduction strengthens the solution by reducing the number of estimated parameters. The two approaches can be combined. It should be stated explicitly that the five-scheme validation and the parameter series share the same data source and processing chain: the WUM precise ephemerides serve as pseudo-observations in one-day-arc dynamic fits, and no station network, elevation cut-off angle or ambiguity resolution is involved. This design ensures that the five schemes are compared on strictly homogeneous data, so the inter-scheme differences reported below isolate the effect of the parameter strategy itself. The five schemes compared in the validation are defined in Table 10, and the detailed POD strategy settings are summarized in Table 11. The benefits for POD are discussed at three levels below.

(1) No accuracy cost and parameter regularization: For brevity, this paper collectively refers to the GEO eclipse windows and the IGSO high-β subset as periods of elevated solar-radiation-pressure modeling error (the stress period below). Across the full-year 2023 five-scheme solutions, the prior-constrained scheme (C) matches the fully unconstrained scheme (A) in within-day fit, orbit prediction, SLR, and inter-center cross-comparison metrics; C/A for this stress period ranges from 0.9977 to 1.0000 (strict stress-period subset; the upper bound for the full stress-period metric is 1.0021, see the Remark column of Table 12). Thus applying the a priori constraints does not degrade accuracy, and the value lies in parameter regularization and the a priori basis (Table 12). (2) Quantified regularization effect (very small reduction): For the three parameters to which priors are applied (2G YSIN, 3I YRAD, 3M-CAST DCOS), the median posterior formal error after applying the priors decreases by 0.042–0.113% relative to fully unconstrained estimation (median arc-wise reduction metric). The normal-equation condition number is essentially unchanged (κ(C/A) = 0.9988–1.0, where κ is the ratio of normal-equation condition numbers), with absolute magnitudes around 10^10^–10^11^, reflecting the near-singular nature of the normal equations. With the σ reconstruction of Table 5 (Section 4.1; σa taken as the sliding-window temporal fluctuation of 0.00413), the recommended σ returns to 2.2–3.3 × 10^−3^ (weights ≈ 10^5^ in 10^−7^ m/s^2^ units; in the dimensionless coefficient units of the POD platform the corresponding weights are of the same order, scaled by the per-satellite F0/M). Reassessed with the reconstructed σ and the combined posterior-σ and condition-number assessment, the regularization effect remains very small (posterior σ reduction and condition number practically unchanged). The prior is therefore a weak-regularization design that achieves parameter regularization without accuracy loss; the weak constraint strength avoids absorbing real signals through overly tight constraints. (3) Value in weak observation-geometry scenarios is left as an outlook: Regional station networks, short arcs and post-maneuver recovery—collectively termed weak-geometry scenarios—have not yet been tested in POD experiments, and their need for a priori constraints and parameter reduction is expected to be stronger. This remains to be verified in future work. Since February 2023 the International Laser Ranging Service (ILRS) has performed laser ranging to all 24 BDS-3 MEO satellites [22]; these centimeter-accuracy SLR data provide a basis for externally validating the a priori models of this paper.

Periods of elevated solar-radiation-pressure modeling error: Eclipse seasons and high-β periods are the intervals with the largest SRP modeling errors, a consensus already reached in existing studies (e.g., Chen et al. [9] handled the D-direction during eclipse seasons, reducing SLR residual standard deviations from 6.2 cm to 3.9 cm (CAST) and from 5.7 cm to 3.1 cm (SECM)). This study provides additional independent empirical evidence: high-β arcs of BDS-3 IGSO satellites C38–C40 show systematic fit residuals of 150–430 cm, positively correlated with |β| (r = 0.527; the median arc RMS is 166.2 cm over the full-year set and 213.0 cm over the high-β subset, and the worst arc reaches 536.6 cm). This confirms that periods of elevated solar-radiation-pressure modeling error are a real, systematic issue, and the value of a priori models during such periods should be evaluated separately. Regarding the degradation mechanism of scheme B (indiscriminate reduction, ECOMC-7): its within-day fit metric B/A = 7.35, one-day extrapolation B/A = 1.97–3.33, SLR off1 B/A = 1.85, and GBM within-arc B/A = 6.89. Mechanistically, order reduction deletes the degrees of freedom that absorb genuine, still-unmodeled SRP signals: once these columns are removed, the orphaned signal is forced into the remaining estimated parameters, distorts their estimates, and propagates into the orbit solution in every arc rather than only under eclipse conditions. The degradation therefore persists throughout the year (one-day B/A is 1.939 inside and 1.980 outside GEO eclipse windows, almost identical) and is orthogonal to the seasonal error sources associated with periods of elevated solar-radiation-pressure modeling error. The two must not be conflated. Validation details are given in Table 12.

### 6.2. Mechanism of the YSIN–β Coupling and Model Limitations

Prange et al. [23] developed an empirical solar radiation pressure model framework for the orbit-normal (ON) attitude mode in which the accelerations are parameterized in a Sun-co-rotating frame by the solar elevation angle β, providing a physical basis for a Y-direction acceleration varying with sin β. The near-perfect linear YSIN–β coupling observed for the GEO group under nominal yaw attitude and the strong YRAD–β coupling for the IGSO group are qualitatively consistent with the picture of a β-modulated systematic Y term in that framework; however, the ON-mode result cannot be directly extrapolated to yaw-stabilized configurations, so the comparison here is only a mechanistic analogy. The reasons why the GEO linear relation can approach *R*^2^ = 0.99 at small β, and the physical origin of the secondary modulation implied by the clustered residual structure, remain to be confirmed through further analysis of the satellite geometry, optical properties, and attitude-control strategy.

### 6.3. Limitations and Future Work

Several limitations remain in this study. The BDS-3 IGSO and BDS-2 MEO groups each contain only three satellites, and the formal uncertainties of the fitted constellation-level coefficients do not account for inter-satellite systematic differences; the corresponding conclusions are therefore sample-specific statements that require validation with a larger sample. The quasi-step change in the GEO group at the end of 2021 and beginning of 2022 has not been attributed to a specific cause; it was absorbed here by relaxing the prior σ rather than through piecewise modeling, and short-period perturbations at the daily resolution were also omitted. The prior σ values were derived from empirical residuals, and the daily residuals exhibit temporal autocorrelation, so the final weights should be retrospectively calibrated by a residual chi-square test during orbit-determination validation. In addition, the C25/C26 subtype split within the SECM group, the formal selection and error budget of the candidate priors for some 2G parameters, and a consistency assessment between the prior values and the parameter products from analysis centers such as CODE and WHU are all left for future work.

## 7. Conclusions

Drawing on the driving-attribution results from five years of daily ECOMC solar radiation pressure parameter series for 42 BDS-2/BDS-3 satellites, this study proposes a two-criterion screening rule combining signal strength (Δ*R*^2^) and inter-satellite consistency (IQR). Differentiated parameter sets and prior-constraint strategies are then built according to orbit group and manufacturer. Two caveats apply to all the conclusions below: the three a priori models are purely empirical statistical models fitted to the observed five-year series rather than physically derived models, and the conclusions for the BDS-3 IGSO and BDS-2 MEO groups each rest on only three satellites, so their direct transfer to newly launched satellites of the same type requires caution. The main conclusions are as follows (definitions of schemes A–E are given in Table 10 and the note to Table 12):

(1) Among 78 group–parameter combinations, 18 are attributable and within-group consistent, 14 are attributable but inconsistent across satellites, and 46 are noise-dominated. Attributable signals concentrate in a small number of parameter–driver combinations and differ by orbit type and manufacturer, providing a basis for differentiated modeling.

(2) For the BDS-2 GEO group, YSIN is almost perfectly linearly coupled to the β angle; the constellation-level prior model YSIN = 0.091604·sin*β* + 0.000548 reaches *R*^2^ = 0.988. The “constant coefficient over five years” assumption holds at first order (sliding-window CV(a) < 5%). The quasi-step from late 2021 to early 2022 (spanning a roughly half-year transition band; see Section 4.1) is conservatively absorbed by inflating the prior σ. We recommend a prior σ of 2.2 × 10^−3^ to 3.3 × 10^−3^ by |β| interval (with σa = 0.00413 taken as the sliding-window temporal fluctuation, in units of 10^−7^ m/s^2^).

(3) For the BDS-3 IGSO group, the YRAD–β relation is best captured by a second-order harmonic form (constellation-level *R*^2^ = 0.750). Leave-one-satellite-out (LOSO) cross-validation gauges the extrapolation performance of the constellation-level coefficients: for C39, β coverage reaches ±78°, yet the predictive *R*^2^ is only 0.24, indicating limited extrapolation capability. With only three in-orbit satellites currently available, per-satellite second-order harmonic priors are recommended as the primary choice, and the constellation-level coefficients should serve only as a degraded fallback for satellites without historical data, with relaxed constraints.

(4) For the BDS-3 MEO-CAST group, DCOS couples a smooth cosβ dependence with an eclipse-season offset. The constellation-level joint model DCOS = 0.01815 − 0.03689·cosβ − 0.00712·E reaches *R*^2^ = 0.814, and the per-satellite eclipse-season offsets are highly consistent (median −0.00733, IQR only 0.0006). C45, which carries the search-and-rescue payload, is an outlier and should be excluded from the constellation-level prior and treated separately.

(5) Differentiated parameter sets and their validation against real precise orbit determination are given for the six orbit groups: (5a) 2G is not reduced (13 parameters + YSIN prior); scheme E is comparable to fully unconstrained estimation. (5b) 2I/2M are not suitable for reduction at present: scheme E is numerically identical to indiscriminate reduction B (E/B = 1.000), with no validated prior available to recover the solution. (5c) 3M-CAST retains the full 13-parameter set with the DCOS prior applied: scheme E (reduction to ECOMC-7) is numerically identical to B (E/B = 1.000). The EM4 control experiment (E plus the M4-based DCOS prior) changes the accuracy metrics by at most 0.6% relative to E, showing that the DCOS prior cannot recover the reduction degradation. (5d) 3I can be reduced to 9 parameters (ECOMC-7 plus freely estimated YCOS and YSIN, with the per-satellite YRAD prior applied); scheme E is close to A and clearly better than B, so the recommendation is supported. (5e) 3M-SECM is reduced only to 10 parameters (YSIN/D2CS/D4CS retained); scheme E is clearly better than B, at a 1.2–3.5-fold cost in fitting accuracy relative to A. (5f) A subtype split at the parameter-attribution level is reported for the first time for SECM group satellites C25 and C26.

Taken together, the full-year 2023 five-scheme validation (Table 12) supports the overall conclusion that prior constraints are neutral and lossless, indiscriminate reduction degrades the solution, and differentiated partial reduction is acceptable. The validation chain also shows that parameter-domain attribution and orbit-domain POD validation are not equivalent: parameter-domain statistics alone cannot guide parameter reduction, and orbit-domain validation is mandatory before any reduced set is adopted in practice.

## Figures and Tables

**Table 1:** Satellite grouping and data overview.

Group	Code	PRN	Satellites	Records
BDS-2 GEO	2G	C01–C05	5	7143
BDS-2 IGSO	2I	C06–C10, C13, C16	7	11,458
BDS-2 MEO	2M	C11, C12, C14	3	4876
BDS-3 IGSO	3I	C38–C40	3	4843
BDS-3 MEO-CAST	3M-CAST	C19–C24, C32, C33, C36, C37, C41, C42, C45, C46	14	22,924
BDS-3 MEO-SECM	3M-SECM	C25–C30, C34, C35, C43, C44	10	16,580
Total	—	—	42	67,824

Note: CAST denotes the China Academy of Space Technology and SECM the Shanghai Engineering Center for Microsatellites, the two manufacturers of the BDS-3 MEO satellites; GEO, IGSO, and MEO denote geostationary Earth orbit, inclined geosynchronous orbit, and medium Earth orbit, respectively.

**Table 2:** Strongest pairwise Pearson correlations of the 13 ECOMC daily parameter estimates within each group (2020–2025).

Group	Satellites	Records	Strongest Pair	r
BDS-2 GEO	5	7143	YSIN–D4CS	−0.7727
BDS-2 IGSO	7	11,458	DSIN–D4CS	−0.7525
BDS-2 MEO	3	4876	BSIN–D2SN	0.4880
BDS-3 IGSO	3	4843	YCOS–YSIN	−0.7274
BDS-3 MEO-CAST	14	22,924	D2CS–D4CS	0.6025
BDS-3 MEO-SECM	10	16,580	BCOS–D2CS	0.7810
Full constellation	42	67,824	BCOS–D2CS	0.7497

**Table 3:** Attribution metrics of the candidate parameters for a priori modeling (three-driver formulation, group-level statistics).

Group	Parameter × Driver	Satellites	Median R^2^_full	Median ΔR^2^	ΔR^2^ IQR	Median Raw r	Modeling Approach
BDS-2 GEO	YSIN × β	5	0.991	0.985	0.002	+0.994	Constellation-level prior (Section 4.1)
BDS-3 IGSO	YRAD × β	3	0.747	0.729	0.213	+0.863	Per-satellite prior (Section 4.2)
BDS-3 MEO-CAST	DCOS × eclipse season	14	0.546	0.527	0.051	−0.730	Constellation-level joint prior (Section 4.3)

**Table 4:** Per-satellite fitted coefficients of YSIN = a·sin*β* + b for the BDS-2 GEO group and five-year sliding-window stability.

Satellite	n	r	R^2^	a ± σa(HAC)	b ± σb(HAC)	CV(a)	Minimum Window R^2^
C01	1428	0.9950	0.9901	0.0918 ± 0.0013	0.0004 ± 0.0003	4.57%	0.9870
C02	1448	0.9927	0.9855	0.0917 ± 0.0012	0.0009 ± 0.0003	4.32%	0.9859
C03	1451	0.9942	0.9884	0.0893 ± 0.0012	0.0008 ± 0.0003	4.42%	0.9857
C04	1375	0.9956	0.9911	0.0927 ± 0.0012	−0.0001 ± 0.0003	4.13%	0.9900
C05	1441	0.9944	0.9889	0.0923 ± 0.0012	0.0009 ± 0.0003	4.54%	0.9854
Constellation	7143	—	0.9884	0.09153 ± 0.00055	0.00057 ± 0.00014	—	—

Note: The constellation-level row uses inverse-variance weighting of the per-satellite coefficients; CV(a) is the coefficient of variation of the slope over a sliding one-year window (step 30 d, 49 windows per satellite); coefficients and σ are in 10^−7^ m/s^2^. σa and σb are Newey–West (Bartlett, m = 20) HAC standard errors (the constellation-level row is combined by inverse-variance weighting of the per-satellite values); the OLS standard errors (constellation-level) are σa = 0.000114 and σb = 0.000031. The constellation-level coefficient a is re-estimated under HAC-error weighting (0.091530), which differs from the OLS-inverse-variance-weighted value in Equation (4) (0.091604) by about 0.1σ, leaving the conclusions unchanged. In the table the coefficients and standard errors are reported with σ rounded to two significant digits and the coefficients aligned to the same decimal place; digits beyond the σ magnitude carry no information and are omitted (the unrounded values are quoted in the text where needed, e.g., the constellation-level σa = 0.000548 above).

**Table 5:** Error budget of the YSIN prior model and recommended prior σ (by |β| interval).

|β| Interval	n	Residual σ	Coefficient-Fluctuation Contribution	Recommended Prior σ	Recommended Weight
0–5°	1143	0.00228	0.00036	0.0023	1.87 × 10^5^
5–10°	1198	0.00213	0.00072	0.0022	1.99 × 10^5^
10–15°	1171	0.00209	0.00107	0.0024	1.82 × 10^5^
15–20°	1566	0.00244	0.00141	0.0028	1.26 × 10^5^
20–23.5°	2065	0.00288	0.00165	0.0033	9.10 × 10^4^
Overall	7143	0.00266	0.00165	0.0031	1.02 × 10^5^

Note: The recommended prior σ = √(σ_interval^2^ + (σa·sin|β|max)^2^), where σa = 0.00413 is the constellation-level average of the 1σ temporal fluctuation of the sliding-window slope (already containing the temporal structure of the quasi-step); σ is in units of 10^−7^ m/s^2^. The HAC slope standard error of 0.000548 (constellation-level row) is given only as a supplementary note on the uncertainty of the coefficient estimate and is not included in the prior σ.

**Table 6:** Comparison of functional forms for YRAD = f(β) (coefficient of determination *R*^2^).

Target	F1 Linear	F2 Piecewise Linear	F3 First-Order Harmonic	F4 Second-Order Harmonic	Decision
C38	0.896	0.897	0.896	0.910	F4
C39	0.474	0.492	0.495	0.585	F4
C40	0.754	0.756	0.755	0.772	F4
Constellation (3 satellites pooled)	0.639	0.642	0.643	0.750	F4

Note: For the constellation-level fit, F4 relative to F1 gives ΔBIC = −1757 and Δ*R*^2^ = +0.111, satisfying the acceptance criterion (ΔBIC < −10 and Δ*R*^2^ > 0.01).

**Table 7:** LOSO cross-validation results of the YRAD prior model.

Left-Out Satellite	Form	Prediction RMSE	Prediction R^2^	Within-Satellite Fit σ	Generalization-Loss Factor
C38	F1 Linear	0.00322	0.624	0.00170	1.90
C38	F4 Second-order harmonic	0.00254	0.766	0.00158	1.60
C39	F1 Linear	0.00377	−0.068	0.00264	1.42
C39	F4 Second-order harmonic	0.00317	0.242	0.00235	1.35
C40	F1 Linear	0.00180	0.753	0.00180	1.00
C40	F4 Second-order harmonic	0.00204	0.682	0.00174	1.18

Note: The generalization-loss factor is the ratio of the held-out prediction RMSE for the left-out satellite to that satellite’s own within-satellite fit σ; a value close to 1 indicates a small extrapolation loss.

**Table 8:** Per-satellite coefficients of the DCOS joint model (continuous cosβ dependence plus eclipse-season offset) for the BDS-3 MEO-CAST group and the constellation-level solutions.

Satellite	c0	c1 (cosβ)	Δc (E)	t(Δc)	R^2^	RMSE
C19	0.015860	−0.035501	−0.007046	−36.8	0.922	0.002517
C20	0.015823	−0.035440	−0.006846	−36.5	0.924	0.002466
C21	0.015773	−0.035335	−0.007114	−38.4	0.927	0.002437
C22	0.015783	−0.035353	−0.007207	−37.7	0.923	0.002509
C23	0.013970	−0.032163	−0.007327	−39.8	0.944	0.002331
C24	0.013937	−0.032104	−0.007448	−42.7	0.949	0.002209
C32	0.016257	−0.034605	−0.007215	−37.2	0.918	0.002553
C33	0.016170	−0.034452	−0.007338	−37.9	0.920	0.002522
C36	0.015015	−0.031916	−0.008010	−39.4	0.934	0.002581
C37	0.015027	−0.031961	−0.007871	−38.1	0.932	0.002614
C41	0.016679	−0.034411	−0.007718	−38.2	0.914	0.002658
C42	0.016635	−0.034356	−0.007647	−37.8	0.914	0.002641
C45	0.038255	−0.058417	−0.006429	−7.3	0.664	0.011202
C46	0.018672	−0.036016	−0.007904	−13.1	0.727	0.006308
Constellation (M4)	0.018150	−0.036889	−0.007116 ± 0.000097	−73.6	0.814	0.004674
Constellation (C1)	0.021826	−0.043583	—	—	0.770	0.005197
Constellation (M1)	−0.006953	—	−0.018572	—	0.423	0.008227

Note: The joint model is DCOS = c0 + c1·cosβ + Δc·E (Equation (6)), with E = 1 denoting the eclipse season (|β| < 13.2°) and E = 0 otherwise; C1 is the pure cosβ model (Δc fixed to zero) and M1 the former piecewise-constant model, for which the c0 column holds the non-eclipse-season constant; the constellation-level rows are combined least-squares fits over all 14 satellites (n = 22,924); coefficients and RMSE are in 10^−7^ m/s^2^. The t values of Δc use OLS standard errors (constellation-level 1σ = 0.000097); the daily residuals are autocorrelated, which inflates the formal significance. The BIC of the constellation-level fits is −220068 (M1), −241122 (C1) and −245978 (M4). The resulting prior, however, is applied only to the 12 consistent satellites: C45 (search-and-rescue payload) and C46 (short series, 1134 records) are excluded and treated separately.

**Table 9:** Recommended parameter sets, a priori constraints and reduction recommendations for the six groups.

Group	Recommended Estimation Set	Reduced Parameters	A Priori Constraint (Verified)	Candidate Prior (to Be Verified)	Exceptional Satellites
BDS-2 GEO	Full 13-parameter set	None (no reduction)	YSIN (Equation (4)); overall σ 0.0031, 0.0022–0.0033 by |β| bin (σa = sliding-window temporal fluctuation 0.00413)	DCOS, BCOS, D4CS, D4SN	— (homogeneous five satellites)
BDS-2 IGSO	Not recommended for reduction for the time being (the pre-validation ECOMC-7 candidate)	(the pre-validation candidates YCOS, YSIN, D2CS, D2SN, D4CS, D4SN)	—	—	—
BDS-2 MEO	Not recommended for reduction for the time being (the pre-validation ECOMC-7 candidate)	(the pre-validation candidates YCOS, YSIN, D2CS, D2SN, D4CS, D4SN)	—	DCOS piecewise (3 satellites, form and σ to be verified)	—
BDS-3 IGSO	ECOMC-9 (ECOMC-7 plus YCOS, YSIN)	D2CS, D2SN, D4CS, D4SN	YRAD per-satellite second-order harmonic (Equation (5)); σ 0.0016–0.0024	—	C39 (high-β coverage ±78°, worst LOSO generalization)
BDS-3 MEO-CAST	Full 13-parameter set (no reduction; reduction to ECOMC-7 not recommended)	None (the EM4 control experiment shows the DCOS prior cannot recover the reduction degradation)	DCOS joint cosβ + eclipse-season-offset model (Equation (7), excluding C45); per-day group-uniform σ in Section 4.3	DRAD × eclipse season	C45 (search-and-rescue payload), C46 (short series, 1134 epochs)
BDS-3 MEO-SECM	10 parameters (ECOMC-7 plus YSIN, D2CS, D4CS)	YCOS, D2SN, D4SN	—	YSIN × β, DCOS × eclipse season (8-satellite subset, excluding C25/C26)	C25, C26 (subtype splitting)

Note: ECOMC-7 denotes the seven-parameter set DRAD, YRAD, BRAD, DCOS, DSIN, BCOS, BSIN, consistent with the ECOM1-7 of Mao et al. [5]; σ and prior-model coefficients in the table are in 10^−7^ m/s^2^; the attribution numbers are the three-driver recomputed values. The exceptional-satellite column marks the applicability limits of each group’s constellation-level/reduction conclusion. Literature comparison: the BDS-2 GEO group is compared with Wang et al. [7], an 8-parameter empirical prior in the DYB frame (2016 POD; SLR RMS < 10 cm, about a 4–5-fold improvement over 5-parameter ECOM). The BDS-2 IGSO/MEO groups are consistent with the ECOM1-7 of Mao et al. [5], whose recommendation is nonetheless segmented (ECOM-7 only for the eclipse and zero-bias arcs, ECOM-5 on the normal arcs) and whose own normal-arc validation degrades accordingly (C08 SLR RMS from 0.06 to 0.12 m); the E-scheme shows that a uniform year-round version of this reduction degrades POD (within-day 5.2-fold/6.8-fold). The BDS-3 IGSO group differs from Li et al.’s [6] ECOMC-8, and the E-scheme is close to free estimation (within-day 1.29-fold) and far better than B. The BDS-3 MEO-CAST group corroborates Chen et al. [9] on eclipse-season D2 over-parameterization, but the E-scheme shows that reduction alone leads to degradation (within-day 7.7-fold); restoring the M4-based DCOS prior on top of the reduced set (scheme EM4) changes all four accuracy metrics (seven metric entries) by at most 0.6% relative to E, so the prior cannot recover the reduction degradation and the group keeps the full 13-parameter set with the DCOS prior applied. The BDS-3 MEO-SECM group does not follow the “remove D2” strategy of Chen et al. [9]; the E-scheme is far better than B (E/B 0.57–0.61) at a 1.2–3.5-fold cost relative to A.

**Table 10:** Definitions and design motivations of the five POD validation schemes (A–E).

Scheme	Definition	Design Motivation
A	Fully unconstrained estimation: all 13 ECOMC parameters freely estimated, no a priori constraint	Reference (free-estimation) baseline
B	Indiscriminate reduction to ECOMC-7 for all groups: with the six columns YCOS, YSIN, D2CS, D2SN, D4CS and D4SN removed	Worst-case bound of parameter reduction under the classical seven-parameter set
C	A plus the verified a priori constraints of Section 4 (2G YSIN, 3I YRAD, 3M-CAST DCOS), introduced as pseudo-observations with weights 1/σ^2^	Tests whether the a priori constraints are neutral in the orbit domain
D	C plus a bias correction applied only to the YSIN of the BDS-2 GEO group (machine-learning residual compensation on the prior mean, σ unchanged; see note)	Tests whether a machine-learning residual compensation on top of the analytic priors brings additional gain
E	The differentiated parameter set of each group as originally suggested at the validation stage (Section 5); for 2I, 2M and 3M-CAST this is the originally suggested reduction to ECOMC-7, whose failure in the validation motivates the final no-reduction recommendation in Table 9	Tests the pre-validation differentiated strategies in the orbit domain

Note: In scheme D a machine-learning bias correction is applied only to the YSIN parameter of the BDS-2 GEO satellites (C01–C05). Scheme D adds to the prior mean of scheme C the daily prediction of a histogram-based gradient-boosting regression model (μ_D = μ_C + dl_pred), while the prior σ is kept unchanged. The model is trained on the residual between the fully unconstrained YSIN estimates and the analytic prior mean, with sin*β*, β, an eclipse flag, the day number within the eclipse season, on-orbit age and time as features. The corrections are precomputed offline into a daily table, and days without a table entry degrade to scheme C. No other group receives this correction: its full-year validation was negative for those groups, and the configuration of scheme D for those groups is therefore the same as that of scheme C.

**Table 11:** POD strategy settings of the five-scheme validation (pseudo-observation design).

Item	Setting
Observations	Pseudo-observations from the WUM precise ephemeris (WUM0MGXFIN SP3), 5 min sampling (289 epochs per arc)
Arc length	1-day (24 h) arc
Estimator	Batch least squares (LSQ)
Estimated parameters	19 per arc: 6 initial state elements + 13 ECOMC solar-radiation-pressure coefficients
Observation weighting	Uniform, σ_obs = 0.03 m
Iterations	N_ITER = 2
Data rejection	None (REJECT_ROUNDS = 0)
Force model	CONFIG_V3 configuration (frozen 4 August 2026)
Software	In-house Python dynamic orbit-determination platform (Python 3.12.13)
Station network/elevation cut-off/ambiguity resolution	Not applicable (pseudo-observation design, no raw GNSS observations)
Design rationale	The pseudo-observation design keeps the five schemes on strictly homogeneous data, so the inter-scheme differences isolate the effect of the parameter strategy itself; the underlying ECOMC parameter series were generated with the ORBFIT module of GAMIT/GLOBK, whereas the five-scheme validation was computed with the in-house platform of this table (two separate software chains; Section 2.1)

**Table 12:** Full-year (2023) five-scheme POD validation results (extrapolation/SLR/GBM/within-day fit).

Metric	Indicator	Value	Remark
Extrapolation (B/A)	1 d	1.97–3.33	Median ratio of six-group RMS-3D (B relative to A, full-year 2023)
Extrapolation (B/A)	12 h	1.95–3.73	Same as above
Extrapolation (C/A)	1 d, period of elevated solar-radiation-pressure modeling error	0.9977–1.0021	GEO eclipse window (DOY 244–288), IGSO high-β (>60°) subsets and adjacent periods (strict stress-period subset measured 0.9977–1.0000)
Extrapolation (E/A)	1 d, 2I/2M/CAST	3.20/2.64/2.91	Originally suggested sets (ECOMC-7) relative to A, numerically identical to B (E/B = 1.000); E-scheme measured
Extrapolation (E/A)	1 d, 3I/SECM/2G	1.13/1.45/1.00	Recommended sets (partial reduction/no reduction) relative to A, clearly better than B; E-scheme measured
Extrapolation (E/A)	12 h, 2G/2I/2M/3I/CAST/SECM	1.000/3.181/3.290/1.161/3.729/1.672	Same basis as the 1 d rows above; E-scheme measured
SLR	off0 B/A	1.40	22 satellites combined (BOTH)
SLR	off1 B/A	1.85	off1 is +1 d extrapolation (adjudication basis)
SLR	Amplification (off1/off0)	1.32	BOTH; per-satellite 0.89–1.74
SLR	off0 E/A (CAST/SECM)	1.369/1.466	E-scheme measured; CAST numerically identical to B (E/B = 1.000)
SLR	off1 E/A (CAST/SECM)	1.958/1.235	Same as above
GBM in-arc	B/A	6.89	GFZ GBM RAP inter-agency difference; median of daily paired ratios; A in-arc median 0.608 m
GBM extrapolation 1 d	B/A	2.51	Same basis as above
GBM in-arc	E/A (CAST/SECM)	8.67/3.38	E-scheme measured: CAST numerically identical to B, SECM clearly better than B (E/B = 0.56)
GBM extrapolation 1 d	E/A (CAST/SECM)	3.22/1.44	Same basis as above; E-scheme measured
Within-day fit	B/A	7.35	1-day (24 h) arc RMS-3D median (A = 70.3 cm, B = 516.9 cm)
Within-day fit	E/A (2I/2M/CAST/3I/SECM/2G)	5.22/6.81/7.65/1.29/3.49/1.00	E-scheme measured: 2I/2M/CAST degrade like B, 3I/SECM partial reduction, 2G retains the full set (E/A = 1.00)

Note: A = fully unconstrained estimation, B = indiscriminate reduction (ECOMC-7 with six columns removed), C = prior-constrained, D = C plus bias correction, E = the differentiated parameter set of each group as originally suggested at the validation stage (Section 5). D is essentially identical to C in the SLR and GBM metrics (D/A ≈ C/A ≈ 1.000); for extrapolation, D coincides with C (C/A = 0.9977–1.0021), so separate D entries are omitted. Scheme E has complete results only under the recommended strategy: for the 2G group E differs from A only by the additional weak YSIN prior and its results are numerically close to A; for the remaining groups only the recommended-strategy indicators are available, and the table lists the corresponding cells. The within-day fit is the median, pooled over all satellite-day arcs of 2023, of the post-fit 3D position-residual RMS from 1-day (24 h) arc least-squares fits of 19 parameters (6 initial state elements plus 13 ECOMC solar-radiation-pressure coefficients) to pseudo-observations taken from the WUM precise ephemeris (5 min sampling, 289 epochs per arc). Extrapolation and within-day fit are median 3D RMS; SLR and GBM are daily/arc-wise aggregates. SLR off0 and off1 denote the zero-extrapolation (same-day arc) and +1-day extrapolation cases, respectively, and BOTH denotes the two manufacturer subsets (CAST and SECM, all 22 SLR-tracked BDS-3 MEO satellites) combined; SLR omits center-of-mass and station tide corrections and is reported as the absolute mean of the residuals. GBM denotes the difference between the in-house orbit and the GFZ GBM orbit product.

## Data Availability

The WUM precise orbit products used as pseudo-observations and the GFZ GBM products used for inter-center comparison are publicly available through the IGS MGEX data center CDDIS (https://cddis.nasa.gov/archive/gnss/products/, accessed on 20 September 2026). The WUM products were downloaded from the Wuhan University MGEX server (ftp://igs.gnsswhu.cn/pub/gnss/products/mgex/, accessed on 20 September 2026). The derived five-year daily ECOMC SRP parameter series (67,824 records for 42 BDS-2/BDS-3 satellites) and the analysis scripts are openly available in a Zenodo repository at https://doi.org/10.5281/zenodo.22045714 (CC BY 4.0).

## Appendix A

This appendix reports the neutrality check of the CAST-group prior model replacement. The full-year 2023 orbit determination of the 14 BDS-3 MEO-CAST satellites was re-run with the joint DCOS prior of Section 4.3 (scheme CM4), and Table A1 compares the resulting group-median accuracy metrics with the original piecewise-prior scheme (C) and the fully unconstrained scheme (A).

**Table A1:** Group-median orbit-accuracy metrics of the full-year 2023 re-run with the M4 DCOS prior (CM4) versus the original piecewise-prior scheme (C) and the fully unconstrained scheme (A) for the 14 BDS-3 MEO-CAST satellites.

Metric	Window	CM4 (M4 Prior)	C (M1 Prior)	A (No Prior)	Δ% (CM4 vs. C)
Time extrapolation	1 d	7.7662	7.7715	7.7662	−0.07
Time extrapolation	12 h	3.8001	3.8089	3.8001	−0.23
SLR residual	off0	1.6766	1.6768	1.6768	−0.01
SLR residual	off1	2.8345	2.8341	2.8347	+0.01
GBM orbit comparison	arc-internal	0.5976	0.5982	0.5970	−0.10
GBM orbit comparison	1 d extrapolated	7.7384	7.7231	7.7086	+0.20
GBM (aligned days)	arc-internal	0.5970	0.5982	0.5970	−0.20
GBM (aligned days)	1 d extrapolated	7.7086	7.7231	7.7086	−0.19
Within-day fit (cm)	1 darcs (4955)	60.4886	60.4888	60.4886	0.00

Note: CM4 denotes the prior-constrained scheme with the M4 DCOS prior, C the original scheme with the piecewise (M1) prior, and A the fully unconstrained scheme. Values are group medians of per-satellite medians over the 14 BDS-3 MEO-CAST satellites (full year 2023; 4955 within-day arcs). Extrapolation and GBM entries are median 3D RMS in meters; SLR entries are RMS in meters (sunlight only, |residual| ≤ 10 m); the within-day fit is the arc-wise 3D RMS in centimeters. Δ% is the relative difference of the group medians, (CM4 − C)/C. All nine metrics differ by no more than 0.24% (C/A ≈ 1.000 throughout), confirming that the neutrality of the prior constraint is insensitive to the functional form of the DCOS prior model.

## References

[1] Beutler G., Brockmann E., Gurtner W., Hugentobler U., Mervart L., Rothacher M., Verdun A. (1994). Extended orbit modeling techniques at the CODE processing center of the International GPS Service for Geodynamics (IGS): Theory and initial results. Manuscr. Geod..

[2] Springer T.A., Beutler G., Rothacher M. (1999). A new solar radiation pressure model for GPS satellites. GPS Solut..

[3] Arnold D., Meindl M., Beutler G., Dach R., Schaer S., Lutz S., Prange L., Sośnica K., Mervart L., Jäggi A. (2015). CODE’s new solar radiation pressure model for GNSS orbit determination. J. Geod..

[4] Bae T.-S., Hong C.-K. (2019). Analysis of parameter correlations of the ECOM solar radiation pressure model for GPS orbit. J. Sens..

[5] Mao Y., Song X.Y., Jia X.L., Ruan R.G. (2017). Analysis about parameters selection strategy of ECOM solar radiation pressure model for BeiDou satellites. Acta Geod. Cartogr. Sin..

[6] Li P.J., Zhang H.W., Xie M.X., Zhao D.F. (2023). A study of solar radiation pressure model in BDS-3 precise orbit determination. Acta Astron. Sin..

[7] Wang C., Guo J., Zhao Q., Liu J. (2019). Empirically derived model of solar radiation pressure for BeiDou GEO satellites. J. Geod..

[8] Chen X., Ge M., Liu Y., He L., Schuh H. (2023). Adapting empirical solar radiation pressure model for BDS-3 medium Earth orbit satellites. GPS Solut..

[9] Chen X., Ge M., Männel B., Schuh H. (2025). Analysis of periodic terms in the DYB frame for the solar radiation pressure model of BDS-3 MEO satellites. GPS Solut..

[10] Duan B., Hugentobler U., Selmke I., Marz S., Killian M., Rott M. (2022). BeiDou satellite radiation force models for precise orbit determination and geodetic applications. IEEE Trans. Aerosp. Electron. Syst..

[11] Wang C., Guo J., Zhao Q., Ge M. (2022). Improving the orbits of the BDS-2 IGSO and MEO satellites with compensating thermal radiation pressure parameters. Remote Sens..

[12] Liu Y., Liu Y., Tian Z., Dai X., Qing Y., Li M. (2019). Impact of ECOM solar radiation pressure models on multi-GNSS ultra-rapid orbit determination. Remote Sens..

[13] Zhao Q., Guo J., Wang C., Lyu Y., Xu X., Yang C., Li J. (2022). Precise orbit determination for BDS satellites. Satell. Navig..

[14] Guo J., Xu X., Zhao Q., Liu J. (2016). Precise orbit determination for quad-constellation satellites at Wuhan University: Strategy, result validation, and comparison. J. Geod..

[15] Montenbruck O., Steigenberger P., Prange L., Deng Z., Zhao Q., Perosanz F., Romero I., Noll C., Stürze A., Weber G. (2017). The Multi-GNSS Experiment (MGEX) of the International GNSS Service (IGS)—Achievements, prospects and challenges. Adv. Space Res..

[16] Guo J., Wang C., Chen G., Xu X., Zhao Q. (2023). BDS-3 precise orbit and clock solution at Wuhan University: Status and improvement. J. Geod..

[17] Li X., Zhu Y., Zheng K., Yuan Y., Liu G., Xiong Y. (2020). Precise orbit and clock products of Galileo, BDS and QZSS from MGEX since 2018: Comparison and PPP validation. Remote Sens..

[18] Montenbruck O., Gill E. (2000). Satellite Orbits: Models, Methods and Applications.

[19] Xia F., Ye S., Chen D., Tang L., Wang C., Ge M., Neitzel F. (2022). Advancing the solar radiation pressure model for BeiDou-3 IGSO satellites. Remote Sens..

[20] Li X., Hu X., Guo R., Tang C., Zhou S., Liu S., Chen J. (2018). Orbit and positioning accuracy for new generation Beidou satellites during the earth eclipsing period. J. Navig..

[21] Rodriguez-Solano C.J., Hugentobler U., Steigenberger P., Allende-Alba G. (2013). Improving the orbits of GPS block IIA satellites during eclipse seasons. Adv. Space Res..

[22] Zajdel R., Nowak A., Sośnica K. (2024). Satellite laser ranging to BeiDou-3 satellites: Initial performance and contribution to orbit model improvement. GPS Solut..

[23] Prange L., Beutler G., Dach R., Arnold D., Schaer S., Jäggi A. (2020). An empirical solar radiation pressure model for satellites moving in the orbit-normal mode. Adv. Space Res..

